# ^1^H-NMR based-metabolomics reveals alterations in the metabolite profiles of chickens infected with ascarids and concurrent histomonosis infection

**DOI:** 10.1186/s13099-023-00584-7

**Published:** 2023-11-17

**Authors:** Oyekunle John Oladosu, Banny Silva Barbosa Correia, Beatrice Grafl, Dieter Liebhart, Cornelia C. Metges, Hanne Christine Bertram, Gürbüz Daş

**Affiliations:** 1 Research Institute for Farm Animal Biology (FBN), Institute of Nutritional Physiology ‘Oskar Kellner’, Wilhelm-Stahl-Allee 2, 18196 Dummerstorf, Germany; 2 Department of Food Science, Aarhus University, Agro Food Park 48, 8200 Aarhus N, Denmark; 3 Clinic for Poultry and Fish Medicine, Department for Farm Animals and Veterinary Public Health, University of Veterinary Medicine, Vienna, Austria

**Keywords:** Blackhead disease, Gastrointestinal infections, Helminths, Infection-induced metabolite alterations, Liver, Metabolome, Plasma, Poultry, Spectroscopy

## Abstract

**Background:**

Gut infections of chickens caused by *Ascaridia galli* and *Heterakis gallinarum* are associated with impaired host performance, particularly in high-performing genotypes. *Heterakis gallinarum* is also a vector of *Histomonas meleagridis* that is often co-involved with ascarid infections. Here, we provide a first insight into the alteration of the chicken plasma and liver metabolome as a result of gastrointestinal nematode infections with concomitant histomonosis. ^1^H nuclear magnetic resonance (^1^H-NMR) based-metabolomics coupled with a bioinformatics analysis was applied to explore the variation in the metabolite profiles of the liver (N = 105) and plasma samples from chickens (N = 108) experimentally infected with *A. galli* and *H. gallinarum* (+*H. meleagridis*). This was compared with uninfected chickens at different weeks post-infection (wpi 2, 4, 6, 10, 14, 18) representing different developmental stages of the worms.

**Results:**

A total of 31 and 54 metabolites were quantified in plasma and aqueous liver extracts, respectively. Statistical analysis showed no significant differences (P > 0.05) in any of the 54 identified liver metabolites between infected and uninfected hens. In contrast, 20 plasma metabolites including, amino acids, sugars, and organic acids showed significantly elevated concentrations in the infected hens (P < 0.05). Alterations of plasma metabolites occurred particularly in wpi 2, 6 and 10, covering the pre-patent period of worm infections. Plasma metabolites with the highest variation at these time points included glutamate, succinate, trimethylamine-*N*-oxide, myo-inositol, and acetate. Differential pathway analysis suggested that infection induced changes in (1) phenylalanine, tyrosine, and tryptophan metabolism, (2) alanine, aspartate and glutamate metabolism; and 3) arginine and proline metabolism (Pathway impact > 0.1 with FDR adjusted P-value < 0.05).

**Conclusion:**

In conclusion, ^1^H-NMR based-metabolomics revealed significant alterations in the plasma metabolome of high performing chickens infected with gut pathogens—*A. galli* and *H. gallinarum.* The alterations suggested upregulation of key metabolic pathways mainly during the patency of infections. This approach extends our understanding of host interactions with gastrointestinal nematodes at the metabolic level.

**Supplementary Information:**

The online version contains supplementary material available at 10.1186/s13099-023-00584-7.

## Background

Infection with gastrointestinal nematode species including *Ascaridia galli* and *Heterakis gallinarum* in chickens is of importance, particularly since these species are re-emerging across Europe due to non-cage housing systems that ease completion of parasite life cycle [1]. The direct effects triggered by these species are associated with impaired host performance [1–3]. These effects are manifested through reduced feed intake, reduced nutrient absorption, eventually leading to potential economic losses [3–6]. Infected birds initiate a rapid immune response within the first weeks of infection through the production of antibodies and activation of the cell-mediated responses that are associated with an effective expulsion of worms [7, 8]. Infection effects are likely dependent on the performance level of the host, such that the performance (e.g., per capita egg mass) of high-performing layer genotypes is considerably penalized during the period of exerting a strong immune response against mixed-nematode infection [3]. Thus, a possible trade-off in the utilisation of metabolic resources required to maintain performance level while developing an effective immune response can be anticipated in nematode-infected chickens.

The reduction in the host’s feed intake [3] and subsequent body weight loss [9] are indicative of the potential changes in the physiology of infected animals. Presently, investigations on the underlying pathophysiological changes induced by gastrointestinal nematode infections in avian species are limited. Studies have focused on such interactions as the activation of the T helper 2 (Th2) immune response including cytokines production [8, 10–12]. However, gut parasites may also interact with the host in other ways, e.g., an interaction between ascarids and endogenous metabolism could be hypothesized. As nematode infections impair host animal performance, the induction of immune responses to expel worms may suggest an interchange in the allocation of metabolic resources between immune function and performance traits [13]. Thus, identifying infection-related alterations in metabolism over time may improve our understanding of host–pathogen interactions and provide insight into potential physiological trade-offs.

Both *A. galli* and *H. gallinarum* often co-infect their common chicken host and have similar direct life cycles, whereby chickens ingest embryonated eggs containing the second or third larval stage from the litter, soil, or contaminated feed and water. The eggs are then hatched in the small intestine within 24 h of ingestion [14, 15]. The larvae of *A. galli* migrate into the superficial mucosal layer of the host small intestine [16] while *H. gallinarum* larvae migrate and develop in the cecum, although with an associated but short tissue phase [17]. Both parasites mature in the lumen of the small intestine and cecum respectively and have a pre-patent period (i.e., time from egg ingestion to the laying of first eggs by adult worms) of between approximately 4–8 weeks [18, 19] after which re-infection can occur, likely earlier with *H. gallinarum*.

*Heterakis gallinarum* is known to be the main vector for the protozoon *Histomonas meleagridis* by harbouring the eggs. Thus, *Heterakis*-infected hens may additionally be exposed to *H. meleagridis,* which can cause severe liver injury to their host [20, 21]. Because of the central role of the liver in metabolism of nutrients and detoxification [22, 23], gastrointestinal nematode infections may further influence liver functions, particularly when histomonosis (syn. blackhead disease) is co-involved in the mixed infections. Depending on the developmental stages of the parasites, they can influence the trade-off between performance and immune defence by altering the metabolism of the host [24–28].

^1^H nuclear magnetic resonance (^1^H-NMR) spectroscopy is a high-throughput analytical method that can be coupled with multivariate analysis to investigate the metabolome of hosts through either biofluids or tissue examination [29]. It could lead to the development of infection signatures through the discovery of novel proxies/biomarkers and, thus enhancing integrated control strategies to improve chicken health and productivity. Given this potential, we hypothesise that the use of ^1^H-NMR based-metabolomics can reveal alterations in the metabolite profile of chickens that may be representative of the trade-off between performance and immune function during mixed nematode infections. In this study, we explore the infection-induced changes in a high-performing commercial line of laying hens. We applied the ^1^H-NMR spectroscopy technique to study the changes in the liver and plasma metabolome at different periods of ascarid infection representing different developmental stages of the worms.

## Results

### Confirmation of ascarid infection in chickens

**Fig. 1:**
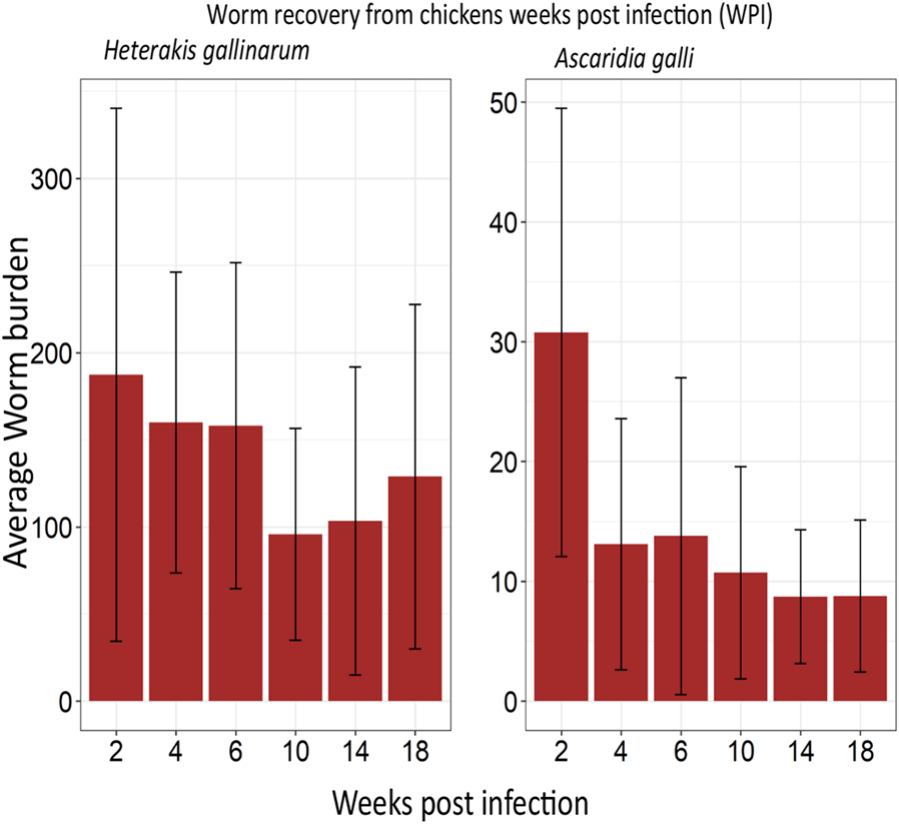
Average worm burdens of hens with *Heterakis gallinarum* and *Ascaridia galli* at different time points. Error bars indicate standard deviation

Infection with both ascarids was confirmed based on the recovery of larval and mature stages of both *A. galli* and *H. gallinarum* from the small intestine and cecum of hens necropsied at different time points after infection with embryonated eggs (Fig. 1). All experimentally infected hens harboured worms, whereas no worms were recovered from the non-infected hens, confirming infection-free status of these control hens.

### Involvement of histomonosis in the infections

**Fig. 2:**
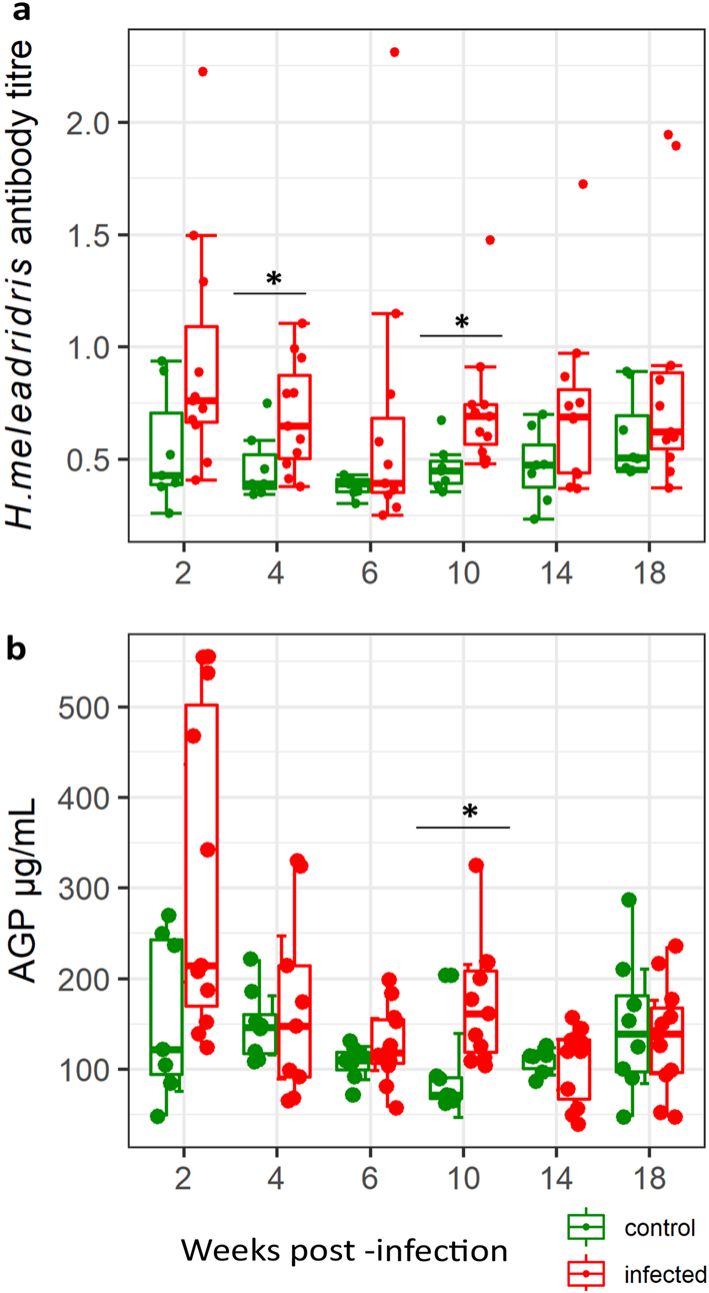
Plasma antibody titres against *Histomonas meleagridis* (**a**) and concentration of plasma alpha (1)-acid glycoprotein (AGP) (**b**) in uninfected (green dots) and infected (red dots) hens throughout experimental weeks. Each dot in the boxplot represents an individual hen. The vertical line inside the boxplots shows the sample median, while the lower and upper end of the box represents the 25th and 75th quantiles respectively. Significant (P < 0.05) differences are marked with (*)

One of the infected hens necropsied at 6^th^ week post-infection (wpi) showed the classical target-like liver lesion associated with histomonosis. Also, the plasma *H. meleagridis* antibody titres were quantified in detail from wpi 2 until the end of the experiment to monitor the co-infection with *H. meleagridis.* About 65% of all the ascarid infected hens were above the predetermined cut-off to be considered positive for histomonosis infection. Moreover, anti-histomonas antibody titre was significantly (P < 0.05) increased in wpi 4 and 10 in infected hens (Fig. 2a), suggesting the presence of *H. meleagridis* in the mixed infections. The concentration of alpha (1)-acid-glycoprotein (AGP) in plasma was also quantified to further determine signs of possible tissue inflammation due to infection. The result showed that the effect of infection was significant (P < 0.05) for the level of AGP at wpi 10 (Fig. 2b). There were no differences in the level of both anti-*H. meleagridis* antibody titres and AGP concentration between infected and uninfected control hens after wpi 10 (P > 0.05).

### Infection-induced alterations in liver and plasma metabolites profiles

**Fig. 3:**
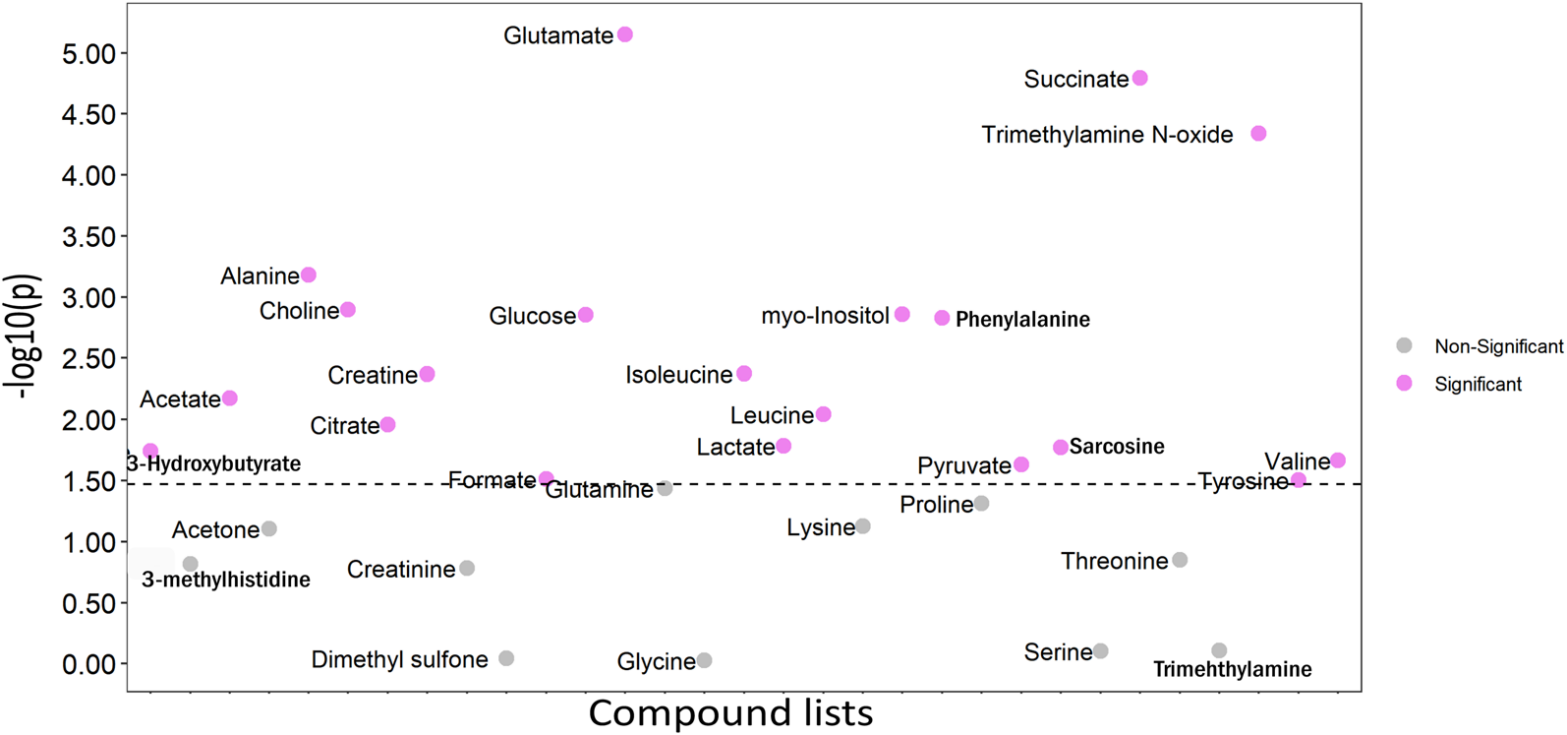
Results of student t-test of plasma metabolite concentrations in nematode infected hens across all wpis. Scatterplot showing compounds selected by two-sample t-tests with an FDR-adjusted *p*-value threshold of 0.05. Pink dots show significant metabolites while the grey dots show non-significant metabolites. Y axis represents the (−)log10 *p*-value

A total of 57 individual metabolites were identified by the Chenomx database in both plasma and liver. In plasma, 31 of the metabolites were present (Additional file 1: Table S1), whereas 54 metabolites were found in the liver samples (Additional file 1: Table S2). The metabolite identifications were verified by the match factor of the NMR spectral signals given by Chenomx software, and by assignment of the signals of 2D spectra. Examples of these assignment details are shown in supporting information (Additional file 4: Figure S2–S10). Univariate analysis with student *t*-test and fold change analysis across the six wpi time-points was used to explore infection-induced changes in the concentration of liver and plasma metabolites. None of the 54 metabolites identified in the liver samples showed significant differences between the infected and uninfected-control groups (Additional file 2: Table S2). However, as shown in Fig. 3, 20 plasma metabolites had significantly (FDR adjusted *P* < 0.05) higher concentrations with fold change (FC) > 1 (Additional file 1: Table S1) in infected samples than samples of the control group. The metabolites glutamate (FC = 1.747), succinate (FC = 1.743), trimethylamine N-oxide (FC = 1.432) and alanine (FC = 1.242) were among the most altered metabolites.

### Time-dependent changes in plasma metabolite concentration due to infection

**Fig. 4:**
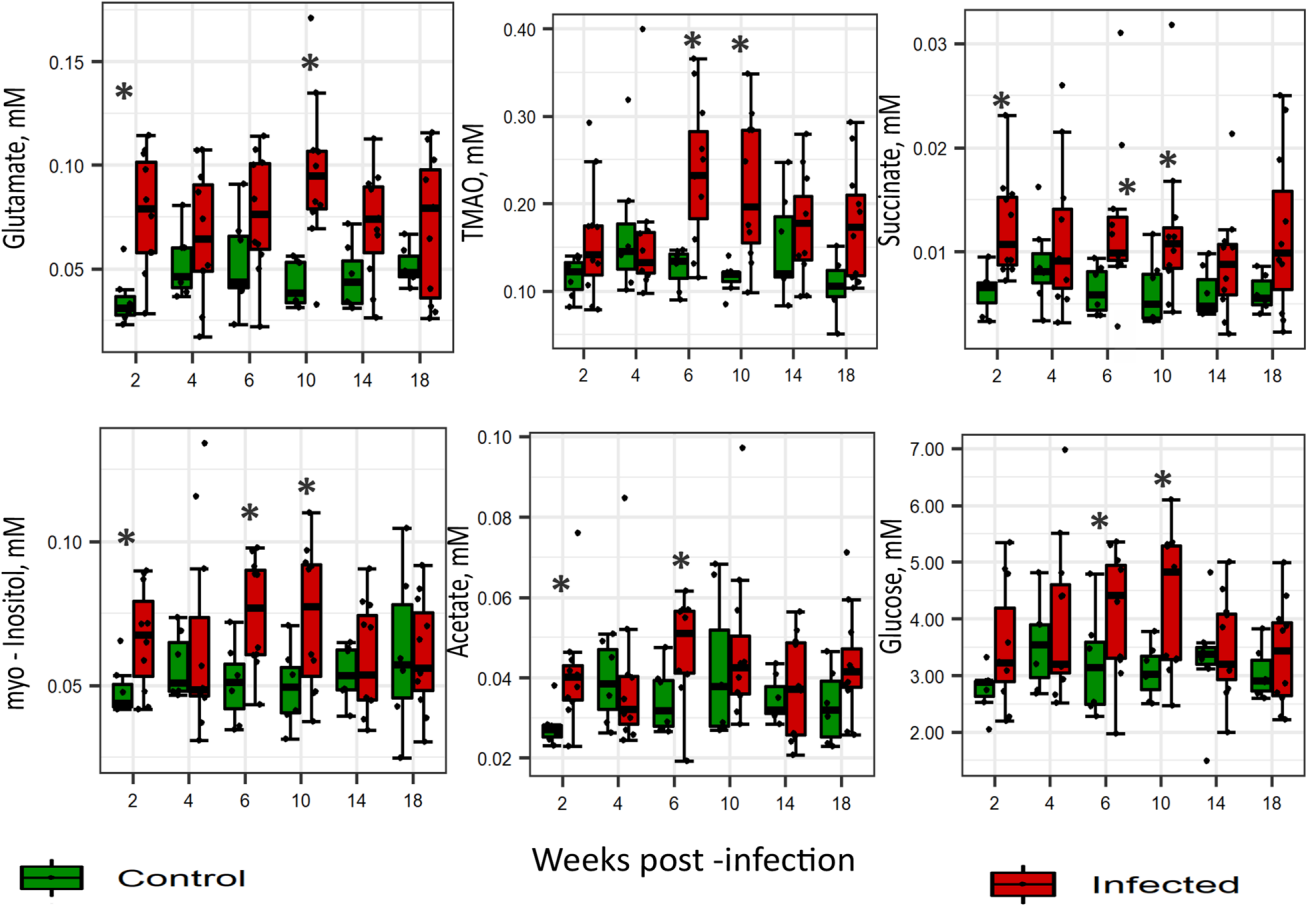
Univariate analysis of selected metabolites showing time—dependent difference between infected (red dots) and control group (green dots). Each dot on the boxplot represents an individual hen. The vertical line inside the boxplots shows the sample median, while the lower and upper end of the box represents the 25th and 75th quantiles respectively. Significant (P < 0.05) differences are marked with (*)

One-way-ANOVA and volcano plot analysis were further performed within each wpi to identify the most important plasma metabolites discriminating infected and non-infected hens at different time points (Fig. 4 & Additional file 3: Figure S1). The analysis identified key time points with significant elevation of specific metabolites in infected hens as compared to non-infected group. Thus, results showed infection-induced changes in plasma metabolites in wpi 2, 6 and 10, while no differences were observed in the concentration of plasma metabolites in wpi 4, 14 and wpi 18. Additional file 3: Figure S1 shows significant differences of metabolite concentration between infected and control groups in wpi 2, 6 and 10. Selected metabolites with significant differences between infected and control hens in at least two time points are visualised in Fig. 4. Citrate, glutamate, succinate, creatinine, leucine, isoleucine, myo-inositol, sarcosine and acetate were the metabolites showing the largest changes in infected samples in wpi 2. In wpi 6, trimethylamine *N*-oxide (TMAO), myo-inositol, creatinine, choline, valine, phenylalanine, acetate, alanine, pyruvate, isoleucine, and succinate were the most altered metabolites. The metabolites most altered in wpi 10 included glutamate, TMAO, 3-hydroxybutyrate, pyruvate, and glucose. At all the time points, metabolites showed significantly higher concentrations in infected compared with non-infected hens. No differences were observed in the concentration of plasma metabolites in wpi 4, 14 and wpi 18.

The hierarchical clustering (Additional file 5: Figure S11) further suggested infection-induced considerable alterations of metabolites at time points up to wpi 10. The pattern of the top 15 significant (based on *t*-test) plasma metabolites within each wpi is presented in Additional file 5: Figure S11.

### Differential alteration of metabolic pathways induced by mixed nematode infection

**Fig. 5:**
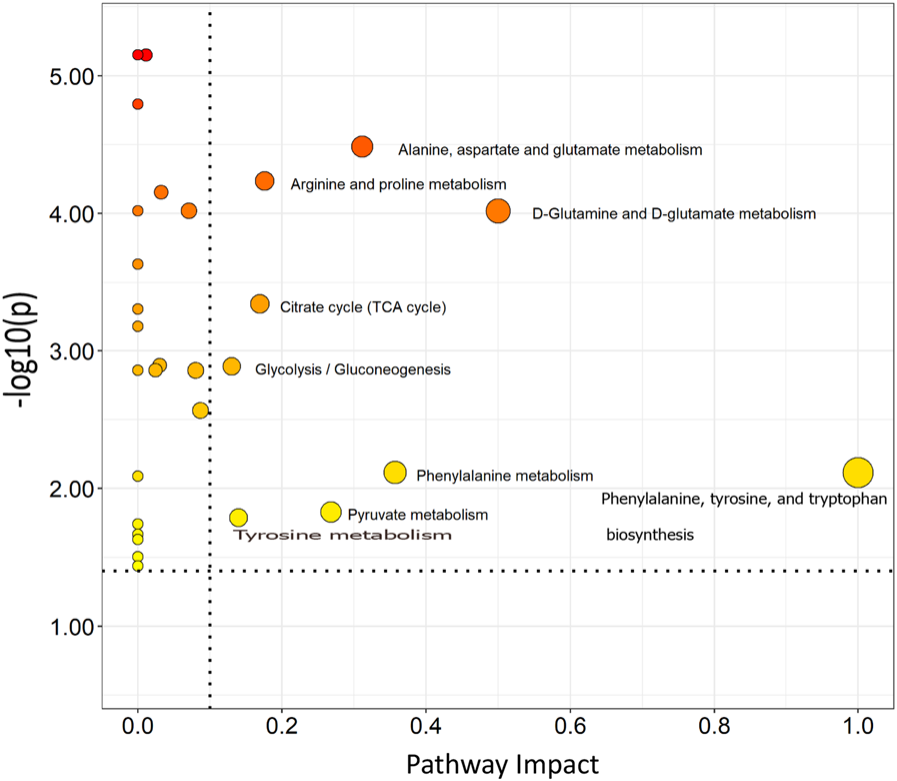
Pathway map. Shows the pathway map of significant metabolites in plasma of laying hens infected with mixed *Ascaridia galli* and *Heterakis gallinarum,* named according to the Additional file 1: Table S1. Size of cycle corresponds to pathway impact with greater size representing greater pathway impact. The darker the colour the greater the −log10(p) values. Pathways with pathway impact > 0.1 and FDR adjusted P-value < 0.05 are labelled and are considered relevant

Figure 5 shows the results of metabolic pathway analysis of significant plasma metabolites. Thirty-three metabolic pathways were found to be involved in the differentiation of control and infected hens. Sixteen pathways had impact values > 0, nine of which had a pathway impact > 0.1 with* P*-value < 0.05 (FDR adjusted). The relevant pathways included (1) Phenylalanine, tyrosine, and tryptophan metabolism; (2) d-Glutamine and d-glutamate metabolism; (3) Phenylalanine metabolism; (4) Alanine, aspartate and glutamate metabolism; (5) Pyruvate metabolism; (6) Arginine and proline metabolism; (7) Tricarboxylic acid cycle; (8) Tyrosine metabolism; (9) Glycolysis/Gluconeogenesis. All 9 pathways were found to contain metabolites that are mainly involved in amino acid and energy metabolism, and which were significantly altered by infection. Three of the identified metabolites including pyruvate, lactate, and glucose were involved in the glycolysis/gluconeogenesis pathway. Similarly, for the alanine, aspartate, glutamate metabolism, the metabolites involved (alanine, citrate, glutamate, succinate, pyruvate, and glutamine) were significantly elevated due to infection. A significant alteration of the citrate cycle was indicated by the infection-induced increase in the concentration of pyruvate, glutamate, alanine, succinate, and citrate. Pathway analysis of significant metabolites across all the weeks post-infections is shown in Additional file 2: Table S3.

## Discussion

This study provides insight into the metabolic alterations that occur in chickens infected with mixed ascarid species. The results presented here show the metabolomic changes induced by mixed infections with two of the most common gastrointestinal nematode species, namely *A. galli* and *H. gallinarum*, as well as concurrent histomonosis infection in a high-performing laying hen genotype. The changes in the plasma metabolites quantified at different time points during infection are an indication of the host-responses occurring at the different developmental stages of worms, particularly during larval penetration of host mucosa (2 wpi), shedding of eggs (> 4 wpi), patency (wpi 4–10) and re-infection (wpi > 10).

We also found that the infection-induced metabolic changes are manifested to a greater extent in the plasma metabolome than the liver metabolome, likely indicating that the plasma metabolome is a better indicator of ascarid infection in chickens. The plasma metabolome showed clear and significant differences in the metabolite profiles of the infected and control groups. Amino acids and sugars were the most strongly altered metabolites and these metabolites were significantly increased in infected hens. One possible reason that could be attributed to the increase of plasma amino acid concentration in infected hens is discrepancy between endogenous protein secretion and re-absorption in the small intestine. Previous studies identified that infection with *A. galli* in chickens was resulted in a large net secretion of nitrogen with a reduced apparent protein absorption in the duodenum of infected chicks [4]. Similarly, reduced activities of proteolytic enzymes (chymotrypsin and trypsin) were observed in the jejunum of ascarid infected chicks [30]. Although, the authors also reported a later reabsorption of protein in the jejunum which may compensate for the losses in the duodenum. Their studies were performed at one time point when worms were at adult stage, hence could not capture the complex dynamics of infection involving both larvae and matured worms. Another possible reason for the increased plasma amino acid concentrations could be the elevated liver protein turnover associated with the acute phase protein response, which occurs as a consequence of infection [31]. Nevertheless, the mechanism of alteration in protein metabolism induced by nematode infections remains elusive and warrants further investigations.

Amino acids are the building blocks for proteins underlying poultry growth and egg formation [32]. A differential upregulation of crucial amino acid pathways such as arginine and proline metabolism and alanine, aspartate, glutamate metabolism was detected in association with the mixed nematode infection. The availability of arginine in the host influences the regulation of the host’s defence mechanism by supporting the activation of T cells and macrophages. Arginine and proline play vital roles in collagen synthesis, contributing to tissue repair and enhancing the function of the intestinal mucosa [33, 34]. Therefore, the upregulation of arginine and proline metabolic pathway may have been a metabolic response to the intestinal inflammation associated with gut infections. Mon et al., 2020 observed such upregulation of arginine and proline metabolism as the most significantly enriched pathway during *Salmonella enteritidis* infection in chickens [35]. They postulated that this upregulation was possibly a metabolic response to the intestinal inflammation caused by *Salmonella* infection.

The plasma glucose concentration remained relatively stable during the initial infection of the hens with mixed nematode species as there were no significant differences between the infected and uninfected groups. However, after a period of re-infection (i.e., 10 wpi), the infected groups exhibited a significantly higher blood glucose level in the plasma. This suggests a potential stress response that influence glucose concentration even though chickens are known to maintain relatively stable blood glucose level [36]. Similar observation was made in chickens infected with *Eimeria acervulina*, where the serum glucose levels were unchanged between infected and uninfected chickens [37]. Collectively, the present findings indicate no conclusive effects of nematode infection on circulating plasma glucose levels.

Another notable alteration due to ascarid infection was the significant increase in the level of plasma TMAO, although it did not seem to contribute to the regulation of any biosynthetic pathways. In mammals, TMAO is formed through liver oxidation of trimethylamine (TMA) by flavin monooxygenase enzyme, and TMA is a product of gut microbial metabolism of compounds such as choline and carnitine [38, 39]. The increased concentrations of both choline and TMAO in this study aligns with this understanding. As TMAO formation is related to both gut microbiota and diet, it has also been shown to be associated with metabolic disorders, systemic inflammation, and cardiovascular disease [39]. In broilers, woody breast myopathy was found to be associated with higher levels of plasma TMAO [40]. Although the role of TMAO in poultry health and diseases is not well understood, it might provide a potential proxy for the identification of parasite-infected individuals. Nonetheless, further studies are necessary to establish the role of TMAO in poultry health.

Other metabolites including sarcosine, creatine, isoleucine, leucine 3- hydroxybutyrate and myo-inositol were also significantly elevated in the plasma due to the mixed infection. Overall, these significant increases reflect considerable metabolic changes that likely indicate adaptation of host metabolism to support physiological demand during gastrointestinal infection. For example, myo-inositol has been associated with immune barrier functions and its deficiency in young grass carp decreases the intestinal immune functions [41]. Whether or not similar role is achieved in poultry remains to be investigated. Similarly, branched chain amino acids play a crucial role in supporting the effective functioning of both the innate and adaptive immune systems and ensuring the integrity of the intestinal mucosa [42]. Given that a robust adaptive immune activation is crucial for an effective worm expulsion [7], we postulate that drastic changes in the branched chain amino acids is likely a metabolic response to the activation of adaptive immune system but, the specific mechanisms and implications of these plasma metabolic alterations in the context of poultry gut infections have not been fully described.

Although a higher number of metabolites could be identified and quantified applying ^1^H-NMR spectroscopy on aqueous liver extracts than on plasma samples, none of the metabolites measured in the liver indicated infection-induced differences. The reason for this lack of changes is elusive. The liver is understandably not the main site of infection for both *A. galli* and *H. gallinarum* but given that liver is a highly metabolic active organ [22, 43] it was hypothesised that liver metabolome could reflect infection-induced changes in host metabolism. The liver metabolome was also studied to further understand concurrent histomonosis infection with ascarid infection. *Heterakis gallinarum* infections are known to predispose their hosts to *H. meleagridis—*a protozoon that potentially causes liver damage in poultry, although with moderate effects in chickens compared to turkeys [44, 45]. Thus, the ascarid-infected hens were additionally investigated for concurrent infection with *H. meleagridis.* One of the hens necropsied in wpi 6 indeed showed typical histomonosis lesion on the liver, otherwise there was no indication of severe liver damage for all hens during post-mortem examination. The antibody against *H. meleagridis* was significantly higher in infected birds in certain wpi (4, 10) and 65% of the infected hens were tested positive for histomonosis based on the cut-off of the serology test [52]. These findings suggest the involvement of concurrent infection with *H. meleagridis.* To gain more insight into the consequences of the mixed infection on liver inflammation, we further measured an acute phase protein, AGP, which is an indication of inflammation [46, 47]. The concentration of AGP as measured in this study was significantly higher in infected birds at the key time points representing worm establishment and patency. Despite this indication of the possible presence of *H. meleagridis,* there was no evidence of severe liver damage during post-mortem examination. This may partly explain the lack of significant changes in the liver metabolome.

To the best of our knowledge no previous studies have examined the alteration of chicken metabolite profiles during nematode infections. While other studies have reported metabolic changes induced by other pathogens, e.g., *Eimeria acervulina* [37]*, Salmonella enteritidis* [35]*,* this study is the first to evaluate the metabolic changes in chickens infected with mixed gastrointestinal nematode species. Future studies should be conducted for broader knowledge, potentially by integrating the application of other metabolomics approaches based on different extracts and other omics technologies (e.g., genomics, transcriptomics, proteomics). Furthermore, a detailed analysis of intestinal tissues as the site of nematode infection could also reveal novel and interesting features that could extend our understanding of the pathogenesis of intestinal parasite infections.

## Conclusions

^1^H-NMR based-metabolomics approach revealed significant alterations in the plasma metabolome of high performing chickens infected with *A. galli* and *H. gallinarum.* The alterations were dependent on both the presence and the patency of infections. Infection upregulated key metabolic pathways. This approach spearheads our understanding of ascarid-host interactions at metabolic level.

## Methods

### Sample collection and study design

A total of 108 laying hens of the Lohmann Brown Plus genotype (LB, N = 108) was used for the infection experiment with *A. galli* and *H. gallinarum.* The laying hens used in this work originated from a previous study [3], where we evaluated the tolerance and resistance of laying hens of different genotypes to nematode infections. We particularly selected this high-performing genotype, as it is more sensitive to the effects of nematode infections. The hens were 24 weeks of age at the start of the experiment as described below. The total number of uninfected control hens was 42 while 66 hens were infected. The pens of infected and non-infected control hens were kept separately in two different rooms to prevent cross-contamination. In each room the LB hens were kept in 3 separate pens in a stocking density of maximum 6 hens per m^2^. Each hen was given a wing-tag to enable repeated measurements on the same individuals over time. The hens were fed a commercial diet (ad libitum), containing 11.2 MJ metabolizable energy, 170 g crude protein and 3.6 g calcium per kg feed [3]. The climatic conditions were optimally regulated using an automatic system to ensure similar lighting, temperature, and aeration across the pens within and between the rooms.

The infection experiment lasted for 18 weeks post infection (wpi), and randomly selected hens from each infection group were necropsied at wpi 2, 4, 6, 10, 14, and 18. Two weeks prior to necropsy hens were randomly selected from their pens (i.e., all pens were sampled with at least one hen), and transferred to individual cages (W 40 × L  45 × H 50 cm). The cages provided equipment for ad libitum water and feed intake of the hens. In each wpi, 11 infected and 7 control birds were necropsied to assess the worm burden as a direct measure of infection intensity. The hens were killed after 3-h feed withdrawal by stunning using a bolt shoot followed by bleeding to death.

Immediately after bleeding to death, blood and liver samples were collected from each bird. Blood was collected in potassium-EDTA treated tubes (Kabe Labortechnik GmbH, Nümbrecht-Elsenroth, Germany) and centrifuged for 20 min at 2500×*g*. The resulting supernatant was stored at − 20 °C for later analysis. The livers were macroscopically examined for typical signs of histomonosis [48]. The liver samples collected from the larger lobe (i.e., right) were snap frozen and stored at − 80 °C until use.

### Experimental infection procedures and diagnosis of infections

The ethics committee for animal experimentation from the Mecklenburg-Western Pomerania State Office for Agriculture, Food Safety and Fishery, Germany, gave approval for the experiment (Permission number AZ.: 7221.3-1-080/16). The experimental procedure for infections followed the guidelines provided by the World Association for the Advancement of Veterinary Parasitology for poultry [49]. Animal handling, care, and necropsies were done by trained and authorised staff members according to the animal welfare rules. Infection material for the experiment was collected from female worms residing in intestines of free-range hens that were naturally infected with ascarids according to the procedure detailed in [50]. At 24 weeks of age, each bird was orally administered 0.4 mL containing a total of 1000 embryonated eggs of two nematodes (*A. galli* and *H. gallinarum*) using a 5-cm oesophageal cannula. The control hens were given a placebo containing 0.4 ml of 0.9% NaCl.

### Determination of worm burden

Worm burdens were quantified from the hens that were necropsied at wpi 2, 4, 6, 10, 14, and 18. The hens were denied access to feed for 3 h prior to necropsies to partly empty the gastrointestinal tract (GIT). The GIT was removed immediately post-mortem, and the small intestine and caecum, the predilection sites of *A*. *galli* and *H. gallinarum*, respectively, were separated. The procedure for opening the intestine has been given in details in [3]. The total number of each of the *A. galli* and *H. gallinarum* worms present in the small intestine and caeca were recorded separately for the respective locations.

### Measurements of plasma anti—Histomonas antibody and alpha (1)-acid glycoprotein

Because *H. gallinarum* is a natural vector for transmission of *H. meleagridis* [45] concomitant histomonosis infections cannot be excluded when the birds are infected with *H. gallinarum*. This also applies to experimental *H. gallinarum* infections, unless heterakis-infected birds are treated against histomonosis [51]. Thus, antibody titres against *H. meleagridis* were measured using an Enzyme Linked Immunosorbent Assay (ELISA) (52) to elucidate whether *H. meleagridis* was involved in the mixed infection. Briefly, ELISA plates were coated with rabbit anti-Histomonas serum at 1:10,000 dilution in carbonate buffer. The plates were treated with blocking buffer after an overnight incubation at 4 °C and a previous wash with PBS-Tween 20 (0.05 per cent PBST). Prior to the next washing step, diluted *H. meleagridis* antigen was added to each well and left for 1 h at room temperature. The plasma samples were then diluted (1:500) with blocking buffer and incubated for another 1 h at room temperature. Each plate included positive and negative control sera obtained from chickens infected experimentally with *H. meleagridis*. Goat anti-chicken IgG-horseradish peroxidase (Southern Biotech, Birmingham, AL, USA) was added for 1 h before another wash. A tetramethylbenzidine substrate solution (TMB; Calbiochem, Merck, Vienna, Austria) was used for 15 min in the dark. The optical density was measured at a wavelength of 450 nm. A predetermined the cut-off OD value of 0.54 nm suggested by Windisch & Hess, 2009 [52] was applied to differentiate birds tested for negative and positive histomonosis.

As *H. meleagridis* can induce damage on the caecal and liver tissues, an acute-phase protein, alpha (1)-acid glycoprotein (AGP) was measured in plasma samples using a commercial ELISA according to the manufacturer’s protocol (Life Diagnostics, West Chester, USA, Catalogue number: LD-AGP-5).

### ***Sample preparation for ***^***1***^***H-NMR spectroscopy***

The plasma samples were thawed on ice and filtered. Prior to filtering, empty filter tubes were initially rinsed with 500 µL of distilled water and centrifuged 3 times for (10,000 × 24 °C × 10 min). An aliquot of 500 µL each of plasma samples was transferred to filter tubes and centrifuged (14,000 × 4 °C × 120 min). For each filtered sample, 350 µL of phosphate buffer (deuterium oxide phosphate buffer 0.10 M, pH = 7.4 containing 3-(trimethylsilyl)-propionic-2,2,3,3-d4 acid, sodium salt (TSP d4) 0.04% and 0.04% of sodium azide) was added to an Eppendorf tube and 350 µL of filtered plasma was added. The tubes were mixed for 2 min at 350 rpm at a table mixer and 600 µL of the solution was transferred to a 5 mm NMR tube.

The frozen liver samples were extracted prior to NMR analysis. The samples were first lyophilized, and 20 mg of lyophilized tissue was weighed, 300 µL of ice-cold methanol (MeOH) was added to samples and whirl mixed thoroughly. Samples were placed on ice for 10 min. A 300 µL of ice-cold water was then added to samples, mixed thoroughly, and placed in 4-degree refrigerator overnight for separation.

Samples were then centrifuged for 30 min × 1400 at 4 °C. The upper MeOH phase was transferred to a new Eppendorf tube and dried for approximately 3 h. Extracted samples were re-dissolved in 575 µL of phosphate buffer (deuterium oxide phosphate buffer 0.10 M, pH = 7.4) and 25 µL of D_2_O with 3-(trimethylsilyl)-propionic-2,2,3,3-d4 acid, sodium salt (TSP d4) 0.05% were added, 550 µL was transferred to NMR tube containing.

### ^***1***^***H-NMR spectrum acquisition***

NMR spectroscopy was conducted at 310 K on a 14 T Bruker Avance III spectrometer (Bruker BioSpin, Rheinstetten, Germany) equipped with a 5 mm TXI probe head with gradients, automated tuning, and matching accessory (ATMATM), BCU-I for the regulation of temperature, and SampleJet robot cooling system set to 5 °C as a sample changer. The ^1^H NMR spectra were acquired using NOESY pre-saturation pulse sequence (Bruker 1D noesygppr1d pulse sequence), 64 K data points, spectral width of 20 ppm, acquisition time of 2.75 s, a recycle delay of 4 s, a relaxation delay of 5*T1 (19 s for blood, 26 s for liver), and 64 scans. 2D NMR experiments (JRES, ^1^H-^13^C HSQC, and ^1^H-^1^H COSY) were performed on selected samples.

The free induction decays (FIDs) were multiplied by a 0.3 Hz exponential function prior to Fourier transform. Phase and baseline corrections were carried out, and the reference standard Trimethylsilylpropanoic acid (TMSP-d4) signal was adjusted to δ 0.00. The 1D spectra were assigned using Chenomix database values, and the 2D NMR spectra.

For quantification of individual metabolites, processed spectra were imported into the Chenomx NMRSuite VX software and metabolite peaks were quantified relative to the area of TMSP-d4 signal. The Chenomx library considers metabolite information such as number of protons and molecular weight. The processing method was set with pH 7.4 and with TMSP-d4 concentrations of 0.41 mM in plasma samples, and 0.1 mM in liver samples.

### Statistical analysis

Statistical analyses of AGP and histomonas antibody titers data were based on log transformation to correct for heterogeneity of variance and to produce approximately normally distributed data. Transformation was done using a natural logarithm function [Ln (y) = ln (y + 1)]. The data were analysed with one-way ANOVA using the R software version 4.1.2 [53].

For the metabolomics data, statistical significance was determined using student t-test with FDR adjustment. Data from all wpi were either pooled or analysed separately within each wpi to examine the effects of infections on metabolite concentrations. One sample with outlier metabolite results was removed from the plasma metabolite data. Differences were considered significant when p < 0.05 and a tendency for significant difference was declared when *0.05* < *p* ≤ *0.10.*

MetaboAnalyst software (http://www.metaboanalyst.ca) was employed for univariate, hierarchical clustering and pathway enrichment analysis of the metabolomics data, and analyses were conducted separately for plasma and liver data, respectively. A hierarchical clustering heatmap showing group averages with Euclidean distance measures and ward linkage was constructed to explore the patterns among the metabolites between the two groups within each wpi. Pareto-scaling normalisation (i.e. normalised based on mean-centered and divided by the square root of the standard deviation of each variable) was applied to metabolite data before analysis. For pathway analysis, data were cross-listed with the pathways in the *Gallus gallus* Kyoto Encyclopedia of Genes and Genomes (KEGG) pathway library. A global test was selected for the enrichment analysis method, while topology analysis was based on relative-betweenness centrality and scatter plot (testing significant features) was chosen for visualisation method.

### Supplementary Information

**Additional file 1: Table S1.** Univariate analysis of plasma metabolites across all the weeks post-infection. **Table S2.** Univariate analysis of liver metabolites across all the weeks post-infection

**Additional file 2: Table S3.** Pathway analysis of significant metabolites across all weeks post-infections

**Additional file 3: Figure S1**. Volcano plot analysis (fold change > 1 and p-value < 0.05) of plasma metabolites at showing significantly higher metabolites in infected hens in wpi 2, 6 and 10.

**Additional file 4: Figure S2.** NMR spectral signal of Trimethylamine-N-oxide from database shown in one-dimensional experiment (1D) data (A) and in two-dimensional (2D) experiment data (B); and from plasma sample of nematode infected hens during wpi 18 shown in 1D experiment (C) and in 2D experiment (D); X axis represents the ^1^H chemical shift in ppm. The blue colour represents the fitting obtained from Chenomx database, the black line represents the real sample, and the Match Factor is given in percentage (C). Frequency 1 (F1 ppm) represents the ^13^C chemical shift in ppm, while the frequency 2 (F2 ppm) represents the ^1^H chemical shift in ppm (B, D). **Figure S3.** NMR spectral signal of Dimethyl sulfone from database shown in one-dimensional experiment (1D) data (A) and in two-dimensional (2D) experiment data (B); and from plasma sample of nematode infected hens during wpi 18 shown in 1D experiment (C) and in 2D experiment (D); X axis represents the 1H chemical shift in ppm. The blue colour represents the fitting obtained from Chenomx database, the black line represents the real sample, and the Match Factor is given in percentage (C). Frequency 1 (F1 ppm) represents the 13C chemical shift in ppm, while the frequency 2 (F2 ppm) represents the 1H chemical shift in ppm (B, D). **Figure S4.** NMR spectral signal of Myo-inositol from database shown in one-dimensional experiment data (1D) (A) and in two-dimensional (2D) experiment data (B); and from plasma sample of nematode infected hens during wpi 18 shown in 1D experiment (C) and in 2D experiment (D); X axis represents the 1H chemical shift in ppm. The blue colour represents the fitting obtained from Chenomx database, the black line represents the real sample, and the Match Factor is given in percentage (C). Frequency 1 (F1 ppm) represents the 13C chemical shift in ppm, while the frequency 2 (F2 ppm) represents the 1H chemical shift in ppm (B, D). **Figure S5.** NMR spectral signal of Serine from database shown in one-dimensional experiment data (1D) (A) and in two-dimensional (2D) experiment data (B); and from plasma sample of nematode infected hens during wpi 18 shown in 1D experiment (C) and in 2D experiment (D); X axis represents the 1H chemical shift in ppm. The blue colour represents the fitting obtained from Chenomx database, the black line represents the real sample, and the Match Factor is given in percentage (c). Frequency 1 (F1 ppm) represents the 13C chemical shift in ppm, while the frequency 2 (F2 ppm) represents the 1H chemical shift in ppm (B, D). **Figure S6.** NMR spectral signal of 3-Methylhistidine from database shown in one-dimensional experiment (1D) data (A); and from plasma sample of nematode infected hens during wpi 18 shown in 1D experiment (B); X axis represents the 1H chemical shift in ppm. The blue colour represents the fitting obtained from Chenomx database, the black line represents the real sample, and the Match Factor is given in percentage (B). **Figure S7.** NMR spectral signal of 1,7-Dimethylxanthine from database shown in one-dimensional experiment (1D) data (A); and from liver sample of nematode infected hens during wpi 18 shown in 1D experiment (B); X axis represents the 1H chemical shift in ppm. The blue colour represents the fitting obtained from Chenomx database, the black line represents the real sample, and the Match Factor is given in percentage (B). **Figure S8.** NMR spectral signal of 3-Methylxanthine from database shown in one-dimensional experiment (1D) data (A) and in two-dimensional (2D) experiment data (B); and from liver sample of nematode infected hens during wpi 18 shown in 1D experiment (C) and in 2D experiment (D); X axis represents the 1H chemical shift in ppm. The blue colour represents the fitting obtained from Chenomx database, the black line represents the real sample, and the Match Factor is given in percentage (C). Frequency 1 (F1 ppm) represents the 13C chemical shift in ppm, while the frequency 2 (F2 ppm) represents the 1H chemical shift in ppm (B, D). **Figure S9.** NMR spectral signal of Oxypurinol from database shown in one-dimensional experiment (1D) data (A) and in two-dimensional (2D) experiment data (B); and from liver sample of nematode infected hens during wpi 18 shown in 1D experiment (C) and in 2D experiment (D); X axis represents the 1H chemical shift in ppm. The blue colour represents the fitting of Chenomx database, the black line represents the real sample, and the Match Factor is given in percentage (C). Frequency 1 (F1 ppm) represents the 13C chemical shift in ppm, while the frequency 2 (F2 ppm) represents the 1H chemical shift in ppm (B, D). **Figure S10.** NMR spectral signal of TMAO from database shown in one-dimensional experiment (1D) data (A) and in two-dimensional (2D) experiment data (B); and from liver sample of nematode infected hens during wpi 18 shown in 1D experiment (C) and in 2D experiment (D); X axis represents the 1H chemical shift in ppm. The blue colour represents the fitting of Chenomx database, the black line represents the real sample, and the Match Factor is given in percentage (C). Frequency 1 (F1 ppm) represents the 13C chemical shift in ppm, while the frequency 2 (F2 ppm) represents the 1H chemical shift in ppm (B, D).

**Additional file 5: Figure S11.** Hierarchical clustering analysis of plasma metabolites. The heat map of the top 25 most significant plasma metabolites between control and infected groups in at all wpis. The patterns of each compound (shown in each row) were categorized by Ward’s clustering algorithm and Euclidean distance metrics. Increased and decreased metabolite concentration are given in red and blue, respectively.

## Data Availability

Data have been made available in open access repository (DOI: 10.5281/zenodo.10108935).

## Abbreviations

^1^H-NMR: ^1^H Nuclear magnetic resonance
KEGG: Kyoto Encyclopaedia of Genes and Genomes
AGP: Alpha (1)-acid glycoprotein
Th2: T helper 2
FID: Free induction decay
FDR: False discovery rate
ELISA: Enzyme linked immunosorbent assay
