# Supplementary material for: 1H-NMR based-metabolomics reveals alterations in the metabolite profiles of chickens infected with ascarids and concurrent histomonosis infection
Source: Gut Pathog. 2023 Nov 17;15:56. doi: 10.1186/s13099-023-00584-7 (PMC10655416; doi:10.1186/s13099-023-00584-7)
Supplement: Supplementary file 1 — Additional file 1: Table S1. Univariate analysis of plasma metabolites across all the weeks post-infection. Table S2. Univariate analysis of liver metabolites across all the weeks post-infection [file 13099_2023_584_MOESM1_ESM.docx]

| **Metabolites** | **p.value** | **-LOG10(p)** | **FDR** | **Fold Change** | **log2(FC)** |
| --- | --- | --- | --- | --- | --- |
| Glutamate | 0.001 | 5.152 | 0.005 | 1.747 | 0.805 |
| Succinate | 0.001 | 4.793 | 0.005 | 1.743 | 0.802 |
| Trimethylamine N-oxide | 0.001 | 4.342 | 0.005 | 1.432 | 0.518 |
| Alanine | 0.001 | 3.181 | 0.005 | 1.242 | 0.312 |
| Choline | 0.001 | 2.895 | 0.006 | 1.273 | 0.348 |
| Myo-Inositol | 0.001 | 2.858 | 0.006 | 1.248 | 0.319 |
| Glucose | 0.001 | 2.854 | 0.006 | 1.203 | 0.266 |
| Phenylalanine | 0.001 | 2.828 | 0.006 | 1.204 | 0.268 |
| Isoleucine | 0.004 | 2.373 | 0.013 | 1.239 | 0.309 |
| Creatine | 0.004 | 2.371 | 0.013 | 1.258 | 0.331 |
| Acetate | 0.007 | 2.174 | 0.019 | 1.210 | 0.275 |
| Leucine | 0.009 | 2.043 | 0.023 | 1.210 | 0.275 |
| Citrate | 0.011 | 1.957 | 0.026 | 1.299 | 0.378 |
| Lactate | 0.016 | 1.786 | 0.035 | 1.241 | 0.311 |
| Sarcosine | 0.017 | 1.772 | 0.035 | 1.260 | 0.334 |
| 3-Hydroxybutyrate | 0.018 | 1.742 | 0.035 | 1.366 | 0.450 |
| Valine | 0.022 | 1.666 | 0.039 | 1.179 | 0.237 |
| Pyruvate | 0.023 | 1.630 | 0.040 | 1.165 | 0.220 |
| Formate | 0.031 | 1.513 | 0.048 | 1.144 | 0.194 |
| Tyrosine | 0.031 | 1.506 | 0.048 | 1.164 | 0.219 |
| Glutamine | 0.037 | 1.437 | 0.054 | 1.166 | 0.221 |
| Proline | 0.049 | 1.314 | 0.068 | 1.156 | 0.210 |
| Lysine | 0.075 | 1.127 | 0.101 | 1.174 | 0.231 |
| Acetone | 0.079 | 1.105 | 0.101 | 1.239 | 0.309 |
| Threonine | 0.141 | 0.850 | 0.175 | 1.105 | 0.144 |
| 3-Methylhistidine | 0.153 | 0.817 | 0.182 | 2.043 | 1.030 |
| Creatinine | 0.164 | 0.784 | 0.189 | 1.208 | 0.272 |
| Trimethylamine | 0.784 | 0.106 | 0.841 | 1.031 | 0.044 |
| Serine | 0.787 | 0.104 | 0.841 | 1.015 | 0.022 |
| Dimethyl sulfone | 0.904 | 0.044 | 0.934 | 0.985 | -0.023 |
| Glycine | 0.942 | 0.026 | 0.942 | 0.992 | -0.011 |

**Additional Table S1** Univariate analysis of plasma metabolites across all the weeks post-infection

**Additional Table S2** Univariate analysis of liver metabolites across all the weeks post-infection

| **Metabolites** | **p.value** | **-LOG10(p)** | **FDR** | **Fold Change** | **log2(FC)** |
| --- | --- | --- | --- | --- | --- |
| 1,7-Dimethylxanthine | 0.134 | 0.872 | 0.773 | 0.861 | -0.216 |
| 3-Hydroxybutyrate | 0.385 | 0.414 | 0.773 | 0.933 | -0.101 |
| 3-Methylxanthine | 0.313 | 0.505 | 0.773 | 0.934 | -0.098 |
| 4-Aminobutyrate | 0.325 | 0.488 | 0.773 | 0.947 | -0.079 |
| Acetate | 0.340 | 0.468 | 0.773 | 0.965 | -0.052 |
| Acetone | 0.082 | 1.084 | 0.773 | 1.066 | 0.093 |
| ADP | 0.987 | 0.006 | 0.996 | 0.999 | -0.001 |
| Alanine | 0.853 | 0.069 | 0.939 | 1.008 | 0.012 |
| AMP | 0.335 | 0.476 | 0.773 | 0.959 | -0.061 |
| Aspartate | 0.665 | 0.177 | 0.887 | 1.019 | 0.027 |
| ATP | 0.996 | 0.002 | 0.996 | 1.000 | 0.000 |
| Betaine | 0.786 | 0.105 | 0.920 | 0.986 | -0.021 |
| Choline | 0.499 | 0.302 | 0.807 | 0.958 | -0.062 |
| Creatine | 0.085 | 1.072 | 0.773 | 0.792 | -0.336 |
| Creatinine | 0.230 | 0.638 | 0.773 | 0.942 | -0.086 |
| Dimethyl sulfone | 0.603 | 0.220 | 0.851 | 1.052 | 0.073 |
| Dimethylamine | 0.254 | 0.596 | 0.773 | 0.934 | -0.098 |
| Formate | 0.403 | 0.395 | 0.773 | 0.976 | -0.035 |
| Fumarate | 0.843 | 0.074 | 0.939 | 1.012 | 0.017 |
| Glucose | 0.781 | 0.108 | 0.920 | 0.985 | -0.021 |
| Glutamate | 0.302 | 0.521 | 0.773 | 0.948 | -0.076 |
| Glutamine | 0.731 | 0.136 | 0.901 | 1.027 | 0.039 |
| Glutathione | 0.737 | 0.132 | 0.901 | 0.985 | -0.022 |
| Glycine | 0.450 | 0.347 | 0.773 | 0.945 | -0.082 |
| Glycolate | 0.887 | 0.052 | 0.956 | 0.990 | -0.014 |
| Guanidoacetate | 0.105 | 0.978 | 0.773 | 0.903 | -0.147 |
| Hypoxanthine | 0.351 | 0.454 | 0.773 | 0.964 | -0.053 |
| 3-Methylhistidine | 0.210 | 0.678 | 0.773 | 0.876 | -0.191 |
| Isoleucine | 0.537 | 0.270 | 0.844 | 0.972 | -0.042 |
| Lactate | 0.931 | 0.031 | 0.966 | 0.996 | -0.005 |
| Leucine | 0.317 | 0.499 | 0.773 | 0.959 | -0.061 |
| Lysine | 0.497 | 0.304 | 0.807 | 1.047 | 0.066 |
| Malonate | 0.406 | 0.392 | 0.773 | 0.944 | -0.084 |
| Methanol | 0.601 | 0.221 | 0.851 | 1.325 | 0.406 |
| Methylamine | 0.409 | 0.389 | 0.773 | 1.040 | 0.057 |
| myo-Inositol | 0.200 | 0.700 | 0.773 | 0.951 | -0.072 |
| N,N-Dimethylglycine | 0.603 | 0.219 | 0.851 | 0.967 | -0.048 |
| NAD+ | 0.678 | 0.169 | 0.887 | 0.986 | -0.020 |
| Niacinamide | 0.438 | 0.359 | 0.773 | 0.953 | -0.070 |
| O-Phosphocholine | 0.908 | 0.042 | 0.960 | 0.991 | -0.014 |
| Oxypurinol | 0.727 | 0.138 | 0.901 | 0.988 | -0.018 |
| Phenylalanine | 0.116 | 0.935 | 0.773 | 0.940 | -0.089 |
| Pyruvate | 0.822 | 0.085 | 0.939 | 0.988 | -0.017 |
| Sarcosine | 0.561 | 0.251 | 0.851 | 0.960 | -0.059 |
| Serine | 0.674 | 0.172 | 0.887 | 0.976 | -0.036 |
| Succinate | 0.436 | 0.361 | 0.773 | 0.965 | -0.051 |
| Threonine | 0.204 | 0.690 | 0.773 | 1.107 | 0.147 |
| Trimethylamine | 0.208 | 0.683 | 0.773 | 0.895 | -0.161 |
| Trimethylamine N-oxide | 0.185 | 0.734 | 0.773 | 1.178 | 0.237 |
| Tyrosine | 0.154 | 0.813 | 0.773 | 0.940 | -0.090 |
| UDP-N-Acetylglucosamine | 0.066 | 1.180 | 0.773 | 0.897 | -0.156 |
| UMP | 0.392 | 0.407 | 0.773 | 0.963 | -0.055 |
| Uridine | 0.383 | 0.417 | 0.773 | 0.945 | -0.082 |
| Valine | 0.370 | 0.431 | 0.773 | 0.962 | -0.056 |
