# Supplementary material for: 1H-NMR based-metabolomics reveals alterations in the metabolite profiles of chickens infected with ascarids and concurrent histomonosis infection
Source: Gut Pathog. 2023 Nov 17;15:56. doi: 10.1186/s13099-023-00584-7 (PMC10655416; doi:10.1186/s13099-023-00584-7)
Supplement: Supplementary file 2 — Additional file 2: Table S3. Pathway analysis of significant metabolites across all weeks post-infections [file 13099_2023_584_MOESM2_ESM.docx]

| **Additional Table S3** Pathway analysis of significant metabolites across all weeks post-infections | | | | | | |
| --- | --- | --- | --- | --- | --- | --- |
| **Pathways** | **Total Compound** | **Hits** | **Raw p ≤** | **-LOG10(p)** | **FDR** | **Impact** |
| Phenylalanine, tyrosine and tryptophan biosynthesis | 4 | 2 | 0.008 | 2.116 | 0.011 | 1.000 |
| D-Glutamine and D-glutamate metabolism | 6 | 2 | 0.001 | 4.021 | 0.001 | 0.500 |
| Phenylalanine metabolism | 8 | 2 | 0.008 | 2.116 | 0.011 | 0.357 |
| Alanine, aspartate and glutamate metabolism | 28 | 6 | 0.001 | 4.485 | 0.001 | 0.311 |
| Pyruvate metabolism | 22 | 3 | 0.015 | 1.831 | 0.019 | 0.267 |
| Arginine and proline metabolism | 38 | 4 | 0.005 | 4.235 | 0.001 | 0.176 |
| Citrate cycle (TCA cycle) | 20 | 3 | 0.009 | 3.343 | 0.001 | 0.169 |
| Tyrosine metabolism | 42 | 2 | 0.016 | 1.787 | 0.020 | 0.140 |
| Glycolysis / Gluconeogenesis | 26 | 4 | 0.001 | 2.888 | 0.002 | 0.130 |
| Glycine, serine and threonine metabolism | 34 | 4 | 0.003 | 2.566 | 0.004 | 0.087 |
| Inositol phosphate metabolism | 30 | 1 | 0.001 | 2.858 | 0.002 | 0.080 |
| Arginine biosynthesis | 13 | 2 | 0.001 | 4.021 | 0.001 | 0.071 |
| Glyoxylate and dicarboxylate metabolism | 32 | 6 | 0.001 | 4.157 | 0.001 | 0.032 |
| Glycerophospholipid metabolism | 35 | 1 | 0.001 | 2.895 | 0.002 | 0.030 |
| Phosphatidylinositol signaling system | 28 | 1 | 0.001 | 2.858 | 0.002 | 0.024 |
| Glutathione metabolism | 28 | 1 | 0.001 | 5.152 | 0.001 | 0.011 |
| Histidine metabolism | 16 | 1 | 0.007 | 5.152 | 0.001 | 0.000 |
| Porphyrin and chlorophyll metabolism | 30 | 1 | 0.001 | 5.152 | 0.001 | 0.000 |
| Propanoate metabolism | 23 | 1 | 0.001 | 4.793 | 0.001 | 0.000 |
| Nitrogen metabolism | 6 | 2 | 0.006 | 4.021 | 0.001 | 0.000 |
| Butanoate metabolism | 15 | 3 | 0.001 | 3.632 | 0.001 | 0.000 |
| Aminoacyl-tRNA biosynthesis | 48 | 9 | 0.001 | 3.306 | 0.001 | 0.000 |
| Selenocompound metabolism | 18 | 1 | 0.001 | 3.181 | 0.002 | 0.000 |
| Galactose metabolism | 27 | 1 | 0.001 | 2.858 | 0.002 | 0.000 |
| Ascorbate and aldarate metabolism | 10 | 1 | 0.001 | 2.858 | 0.002 | 0.000 |
| Valine, leucine and isoleucine biosynthesis | 8 | 3 | 0.008 | 2.090 | 0.011 | 0.000 |
| Valine, leucine and isoleucine degradation | 40 | 3 | 0.008 | 2.090 | 0.011 | 0.000 |
| Synthesis and degradation of ketone bodies | 5 | 1 | 0.018 | 1.742 | 0.021 | 0.000 |
| Pantothenate and CoA biosynthesis | 19 | 1 | 0.022 | 1.666 | 0.025 | 0.000 |
| Cysteine and methionine metabolism | 33 | 1 | 0.023 | 1.630 | 0.026 | 0.000 |
| Ubiquinone and other terpenoid-quinone biosynthesis | 9 | 1 | 0.031 | 1.506 | 0.033 | 0.000 |
| Purine metabolism | 62 | 1 | 0.037 | 1.437 | 0.037 | 0.000 |
| Pyrimidine metabolism | 40 | 1 | 0.037 | 1.437 | 0.037 | 0.000 |
