# Supplementary figures and images for: 1H-NMR based-metabolomics reveals alterations in the metabolite profiles of chickens infected with ascarids and concurrent histomonosis infection
Source: Gut Pathog. 2023 Nov 17;15:56. doi: 10.1186/s13099-023-00584-7 (PMC10655416; doi:10.1186/s13099-023-00584-7)

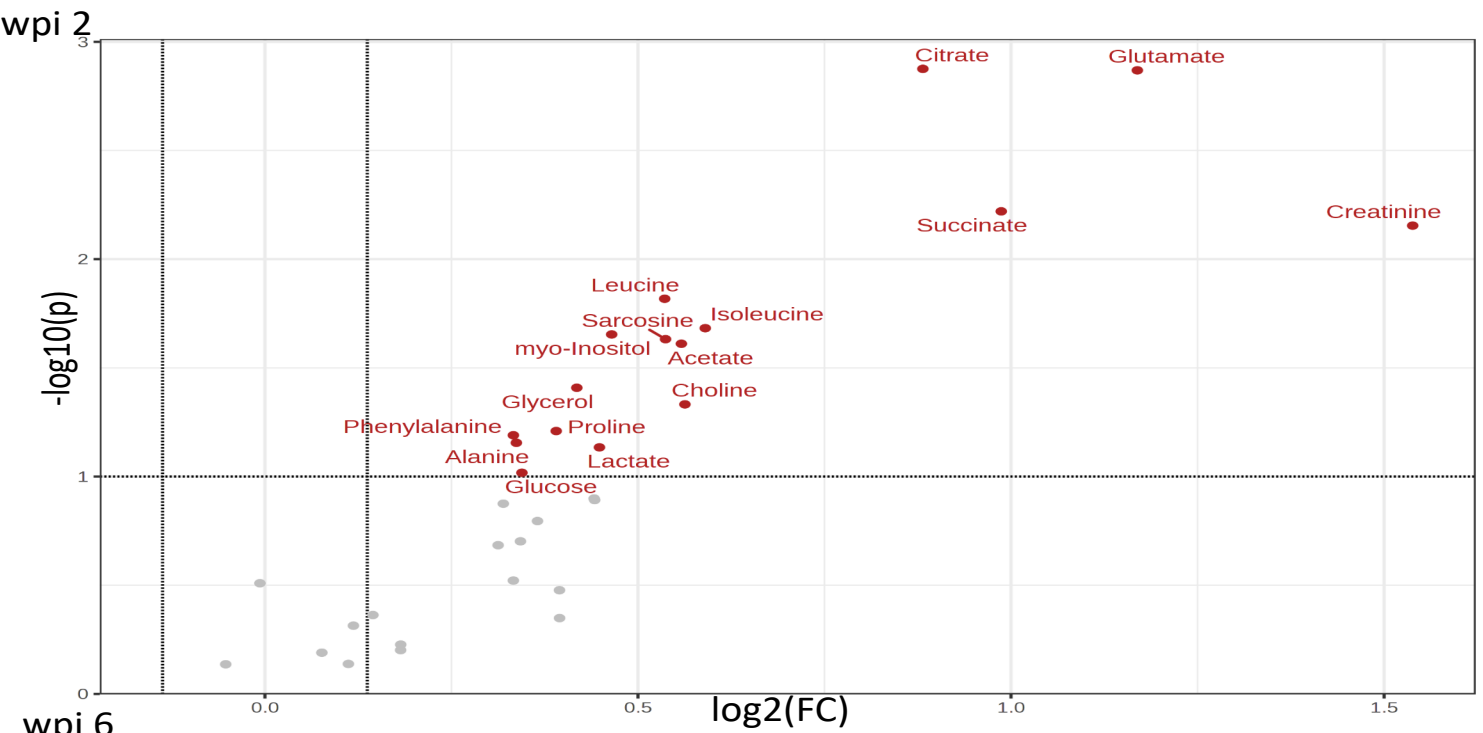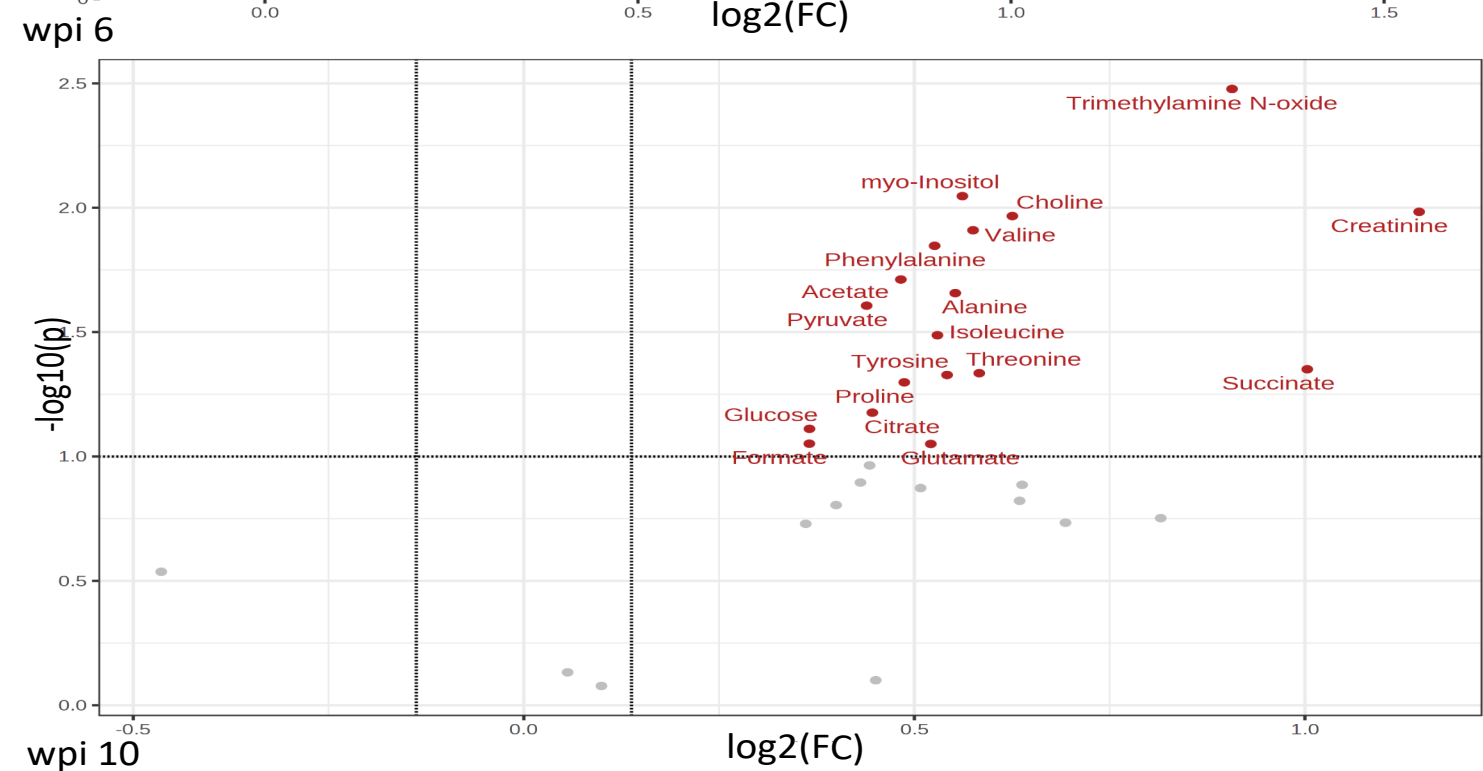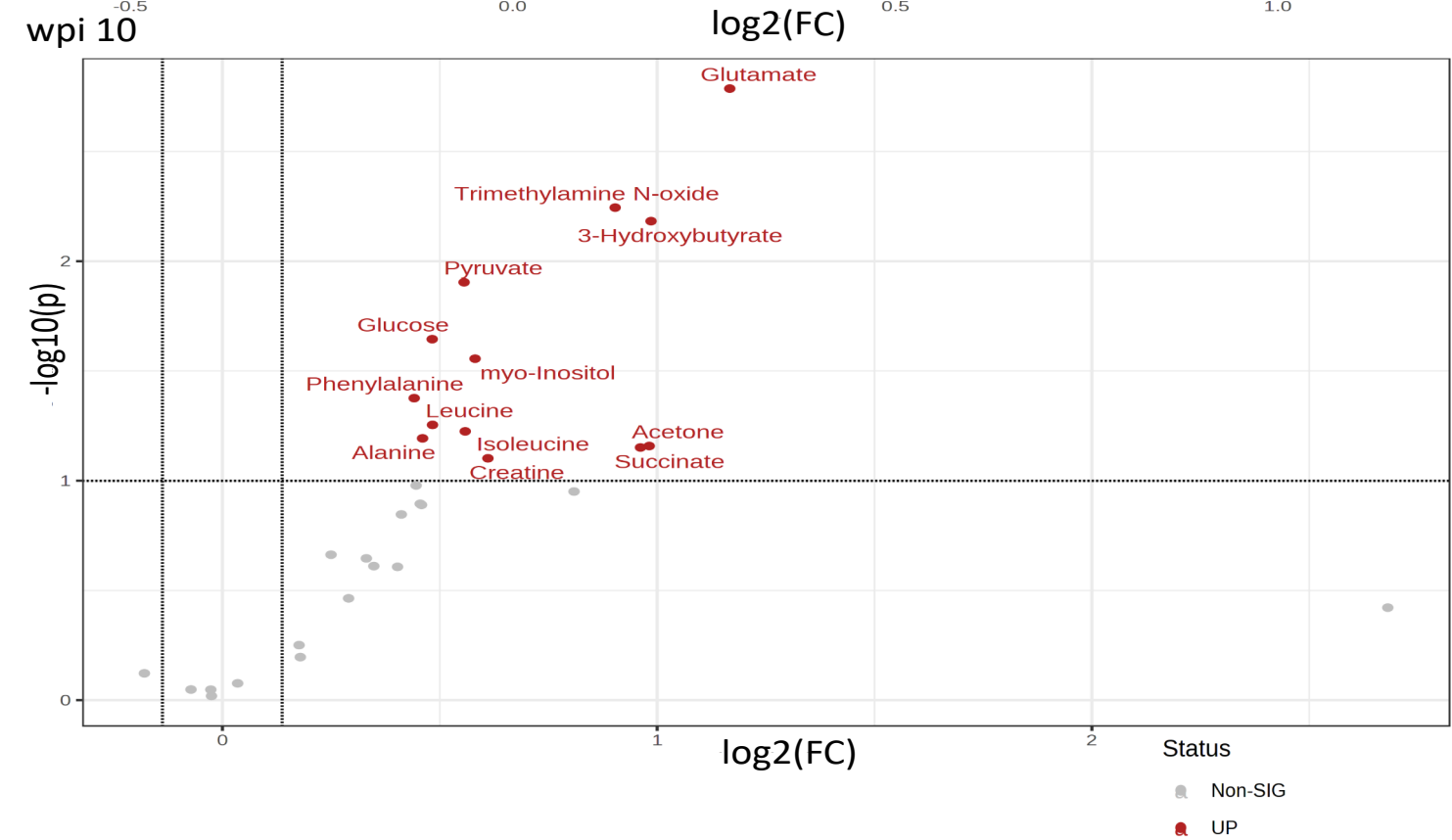

Supplement: Supplementary file 3 — Additional file 3: Figure S1. Volcano plot analysis (fold change > 1 and p-value < 0.05) of plasma metabolites at showing significantly higher metabolites in infected hens in wpi 2, 6 and 10. [file 13099_2023_584_MOESM3_ESM.pdf]

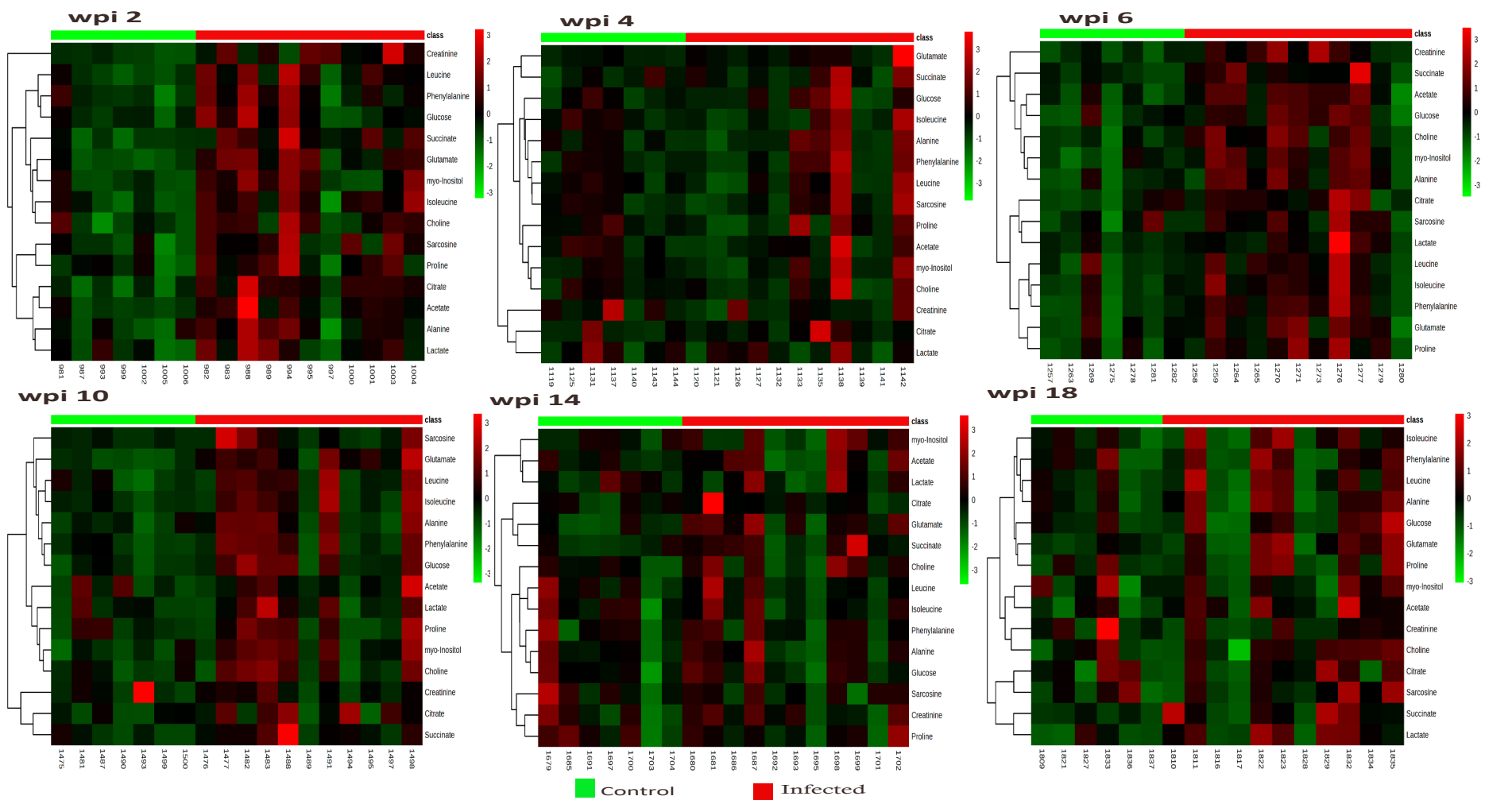

Supplement: Supplementary file 5 — Additional file 5: Figure S11. Hierarchical clustering analysis of plasma metabolites. The heat map of the top 25 most significant plasma metabolites between control and infected groups in at all wpis. The patterns of each compound (shown in each row) were categorized by Ward’s clustering algorithm and Euclidean distance metrics. Increased and decreased metabolite concentration are given in red and blue, respectively. [file 13099_2023_584_MOESM5_ESM.pdf]
