# Supplementary material for: 1H-NMR based-metabolomics reveals alterations in the metabolite profiles of chickens infected with ascarids and concurrent histomonosis infection
Source: Gut Pathog. 2023 Nov 17;15:56. doi: 10.1186/s13099-023-00584-7 (PMC10655416; doi:10.1186/s13099-023-00584-7)
Supplement: Supplementary file 4 — Additional file 4: Figure S2. NMR spectral signal of Trimethylamine-N-oxide from database shown in one-dimensional experiment (1D) data (A) and in two-dimensional (2D) experiment data (B); and from plasma sample of nematode infected hens during wpi 18 shown in 1D experiment (C) and in 2D experiment (D); X axis represents the 1H chemical shift in ppm. The blue colour represents the fitting obtained from Chenomx database, the black line represents the real sample, and the Match Factor is given in percentage (C). Frequency 1 (F1 ppm) represents the 13C chemical shift in ppm, while the frequency 2 (F2 ppm) represents the 1H chemical shift in ppm (B, D). Figure S3. NMR spectral signal of Dimethyl sulfone from database shown in one-dimensional experiment (1D) data (A) and in two-dimensional (2D) experiment data (B); and from plasma sample of nematode infected hens during wpi 18 shown in 1D experiment (C) and in 2D experiment (D); X axis represents the 1H chemical shift in ppm. The blue colour represents the fitting obtained from Chenomx database, the black line represents the real sample, and the Match Factor is given in percentage (C). Frequency 1 (F1 ppm) represents the 13C chemical shift in ppm, while the frequency 2 (F2 ppm) represents the 1H chemical shift in ppm (B, D). Figure S4. NMR spectral signal of Myo-inositol from database shown in one-dimensional experiment data (1D) (A) and in two-dimensional (2D) experiment data (B); and from plasma sample of nematode infected hens during wpi 18 shown in 1D experiment (C) and in 2D experiment (D); X axis represents the 1H chemical shift in ppm. The blue colour represents the fitting obtained from Chenomx database, the black line represents the real sample, and the Match Factor is given in percentage (C). Frequency 1 (F1 ppm) represents the 13C chemical shift in ppm, while the frequency 2 (F2 ppm) represents the 1H chemical shift in ppm (B, D). Figure S5. NMR spectral signal of Serine from database shown in one-dimensional [file 13099_2023_584_MOESM4_ESM.pdf]

Figure S2

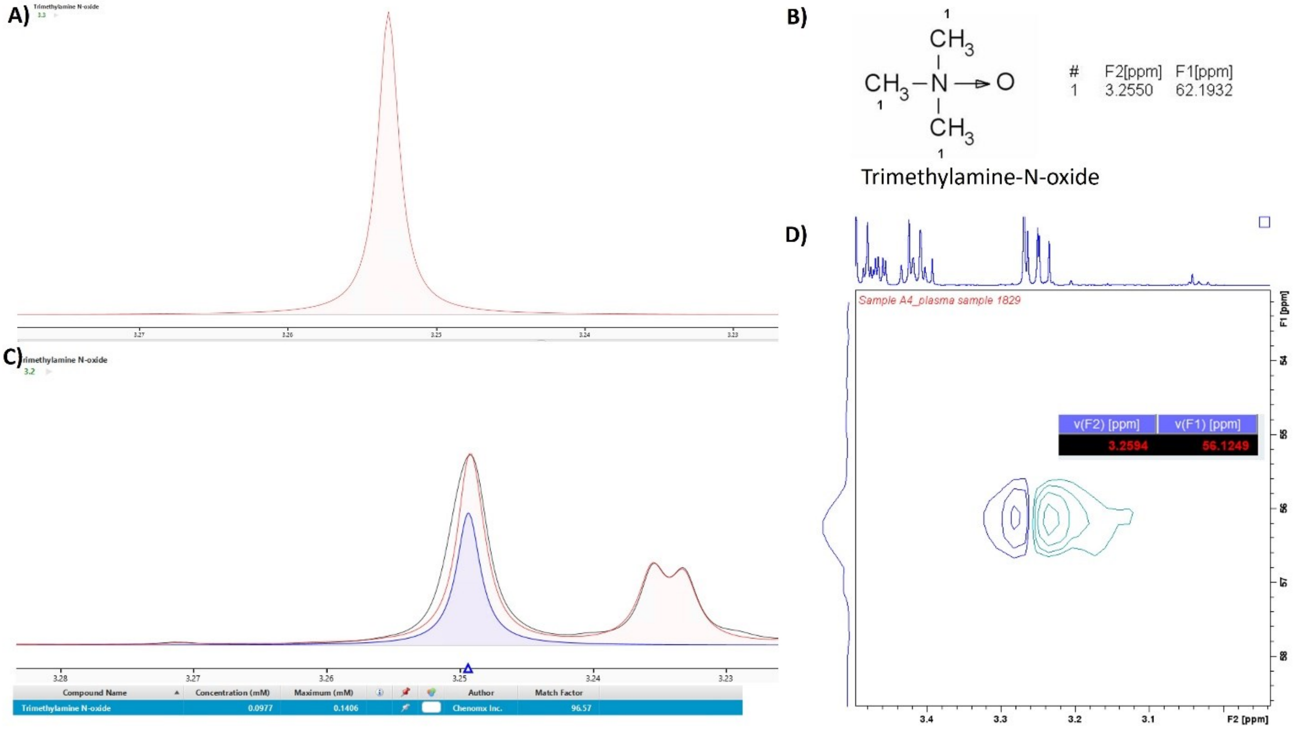

A)
Trimethylamine N-oxide
3.27
3.26
3.25
3.24
3.23
B)
Trimethylamine-N-oxide
F2[ppm] F1[ppm]
1 3.2550 62.1932
C)
Trimethylamine N-oxide
3.28
3.27
3.26
3.25
3.24
3.23
Compound Name
Concentration (mM)
Maximum (mM)
Author
Match Factor
Trimethylamine N-oxide
0.0977
0.1406
Chenomx Inc.
96.57
D)
Sample A4_plasma sample 1829
v(F2) [ppm]
v(F1) [ppm]
3.2594
56.1249
F1 [ppm]
54
55
56
57
58
3.4
3.3
3.2
3.1
F2 [ppm]

Figure S3

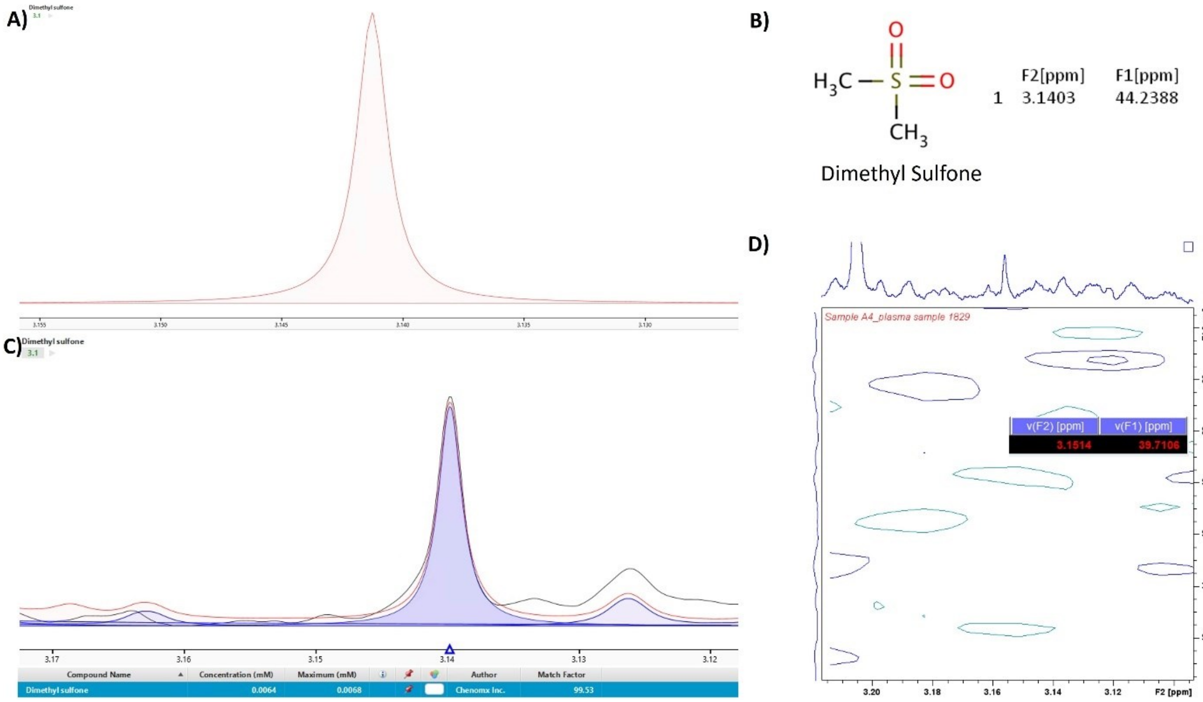
A)
Dimethyl sulfone
3.155
3.150
3.145
3.140
3.135
3.130
B)
Dimethyl Sulfone
F2[ppm]
F1[ppm]
1
3.1403
44.2388
C)
Dimethyl sulfone
3.17
3.16
3.15
3.14
3.13
3.12
Compound Name
Concentration (mM)
Maximum (mM)
Author
Match Factor
Dimethyl sulfone
0.0064
0.0068
Chenomx Inc.
99.53
D)
Sample A4_plasma sample 1829
v(F2) [ppm]
v(F1) [ppm]
3.1514
39.7106
3.20
3.18
3.16
3.14
3.12
F2 [ppm]
F1 [ppm]

Figure S4

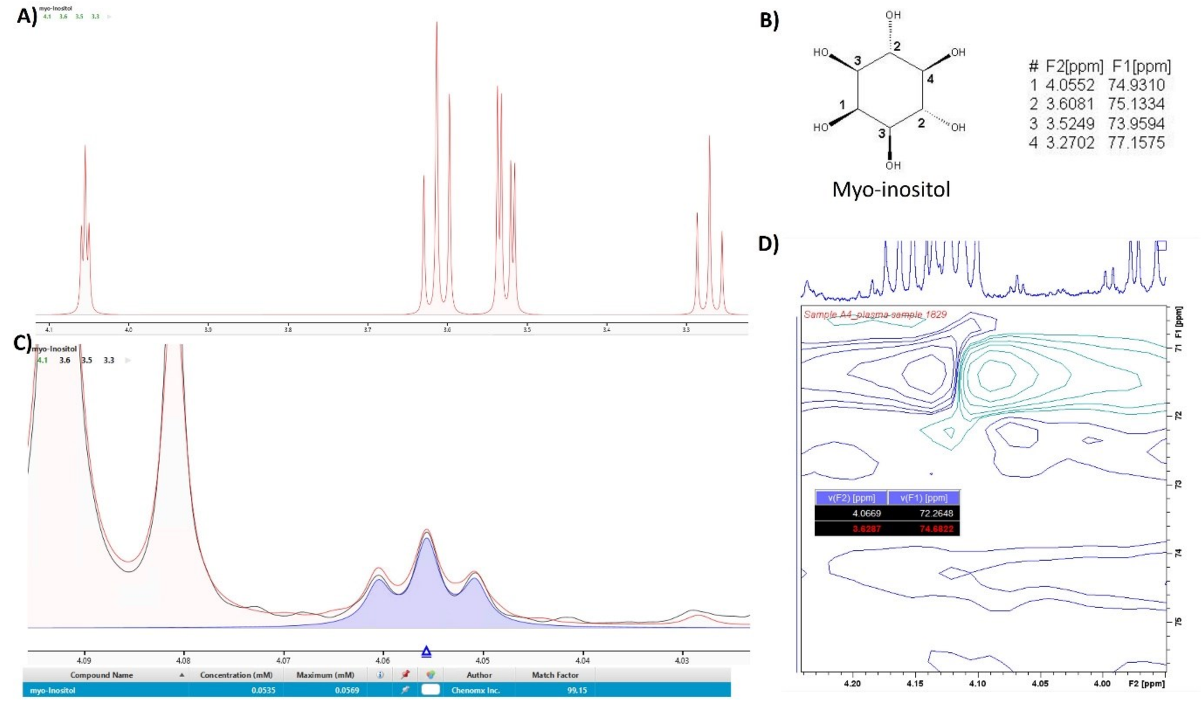
A)
myo-Inositol
4.1 3.6 3.5 3.3
B)
Myo-inositol
F2[ppm] F1[ppm]
1 4.0552 74.9310
2 3.6081 75.1334
3 3.5249 73.9594
4 3.2702 77.1575
C)
myo-Inositol
4.1 3.6 3.5 3.3
4.09 4.08 4.07 4.06 4.05 4.04 4.03
Compound Name
Concentration (mM)
Maximum (mM)
Author
Match Factor
myo-Inositol 0.0535 0.0569 Chenomx Inc. 99.15
D)
Sample A4_plasma sample 1829
v(F2) [ppm] v(F1) [ppm]
4.0669 72.2648
3.6287 74.6822
F1 [ppm]
71 72 73 74 75
4.20 4.15 4.10 4.05 4.00 F2 [ppm]

Figure S5

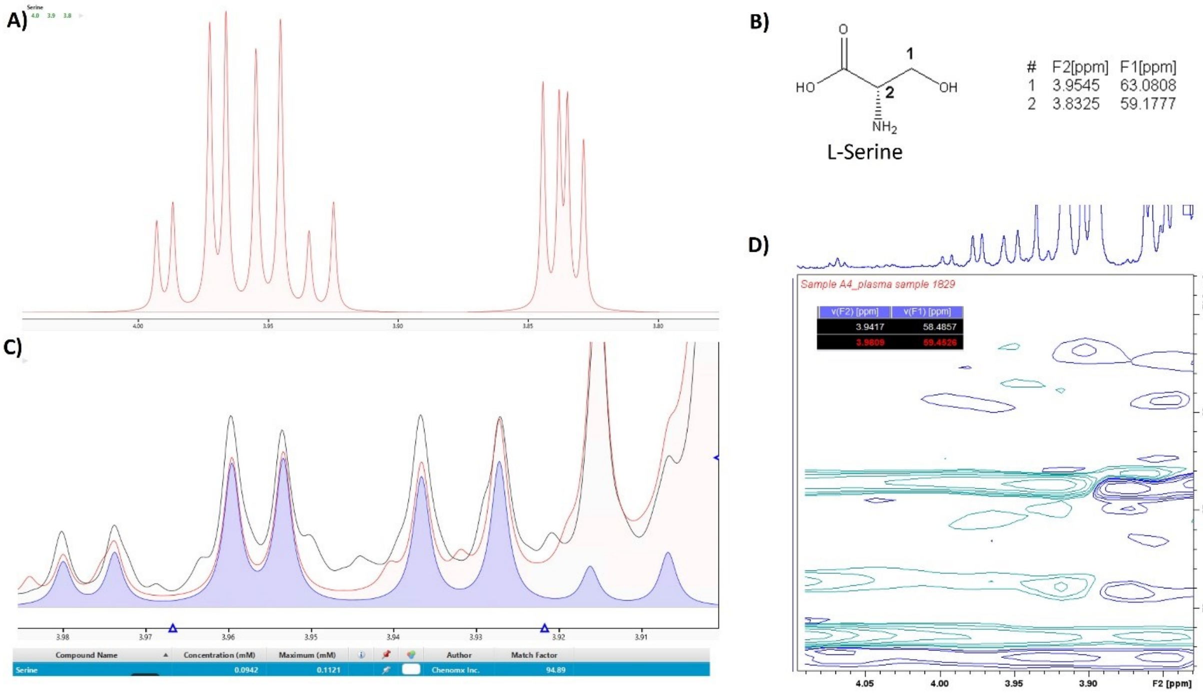
A)
Serine
4.00
3.95
3.90
3.85
3.80
B)
O
1
HO
2
OH
NH_2
L-Serine
F2[ppm] F1[ppm]
1 3.9545 63.0808
2 3.8325 59.1777
C)
3.98
3.97
3.96
3.95
3.94
3.93
3.92
3.91
Compound Name
Concentration (mM)
Maximum (mM)
Author
Match Factor
Serine
0.0942
0.1121
Chenomx Inc.
94.89
D)
Sample A4_plasma sample 1829
v(F2) [ppm]
v(F1) [ppm]
3.9417
58.4857
3.9809
59.4526
F1 [ppm]
60
65
70
4.05
4.00
3.95
3.90
F2 [ppm]

Figure S6

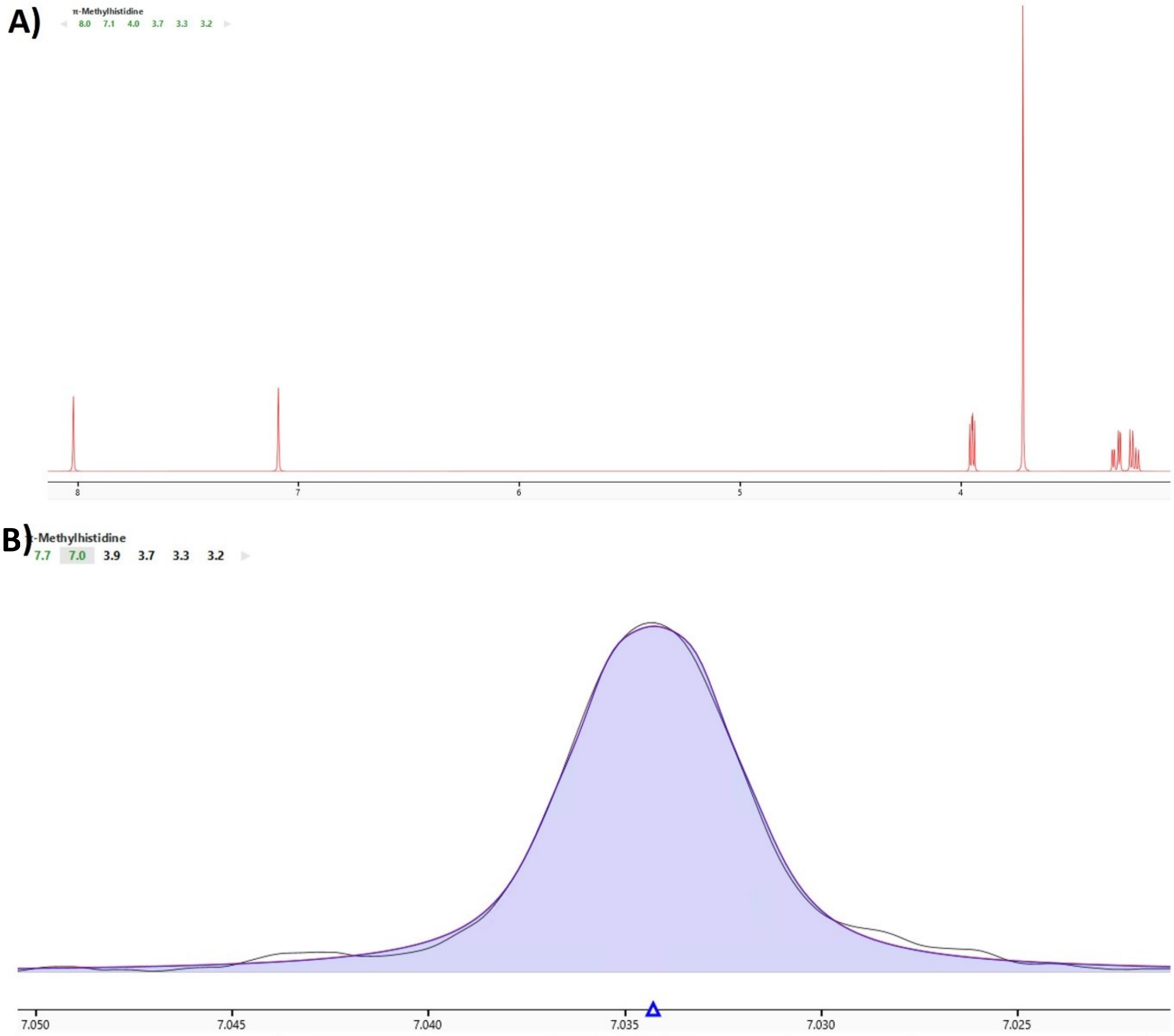

Compound Name	Concentration (mM)	Maximum (mM)				Author	Match Factor
π-Methylhistidine	0.0278	0.0280				Chenomx Inc.	99.92

Figure S7

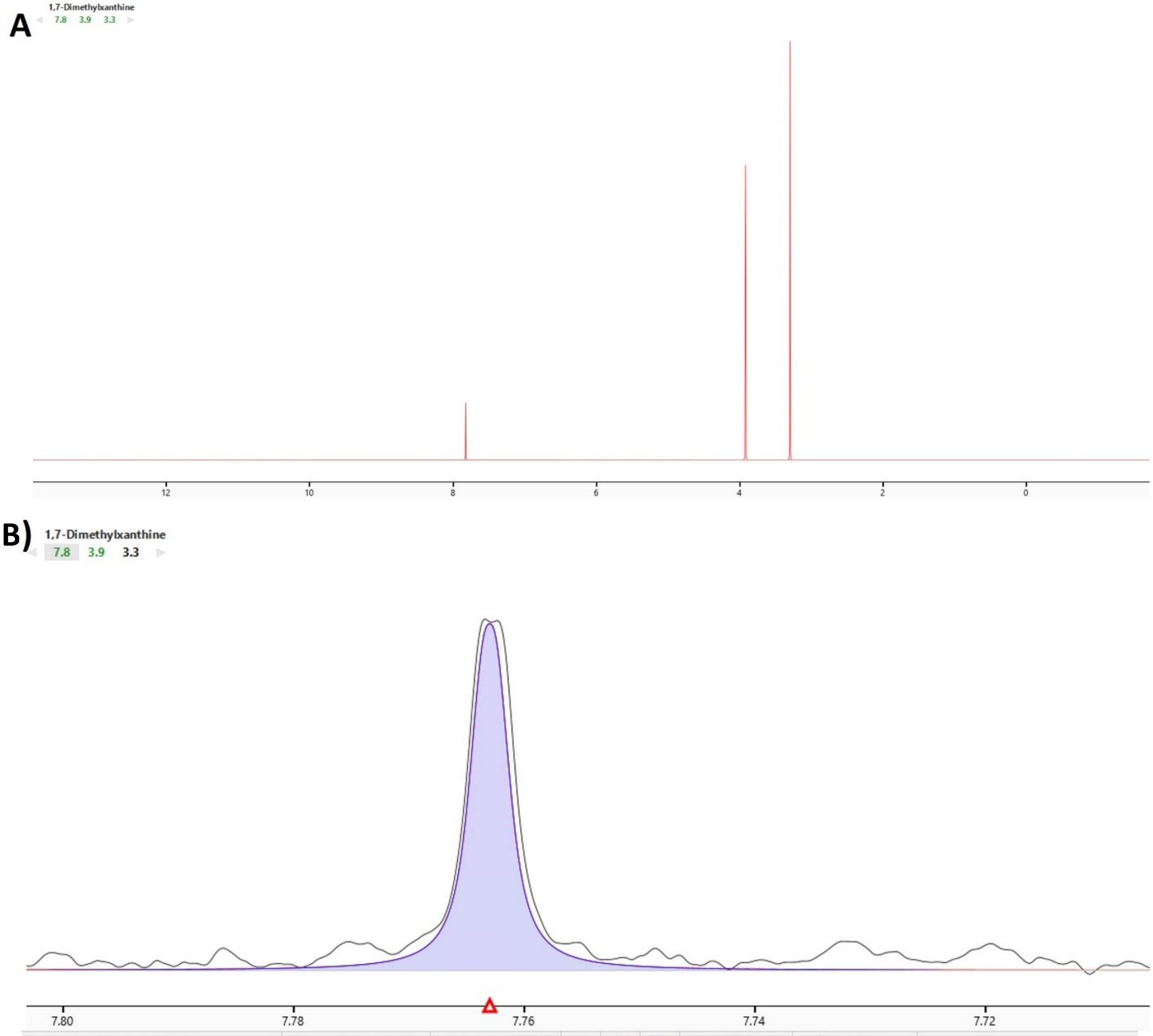

Compound Name	Concentration (mM)	Maximum (mM)				Author	Match Factor
1,7-Dimethylxanthine	0.0367	0.0167				Chenomx Inc.	98.76

Figure S8

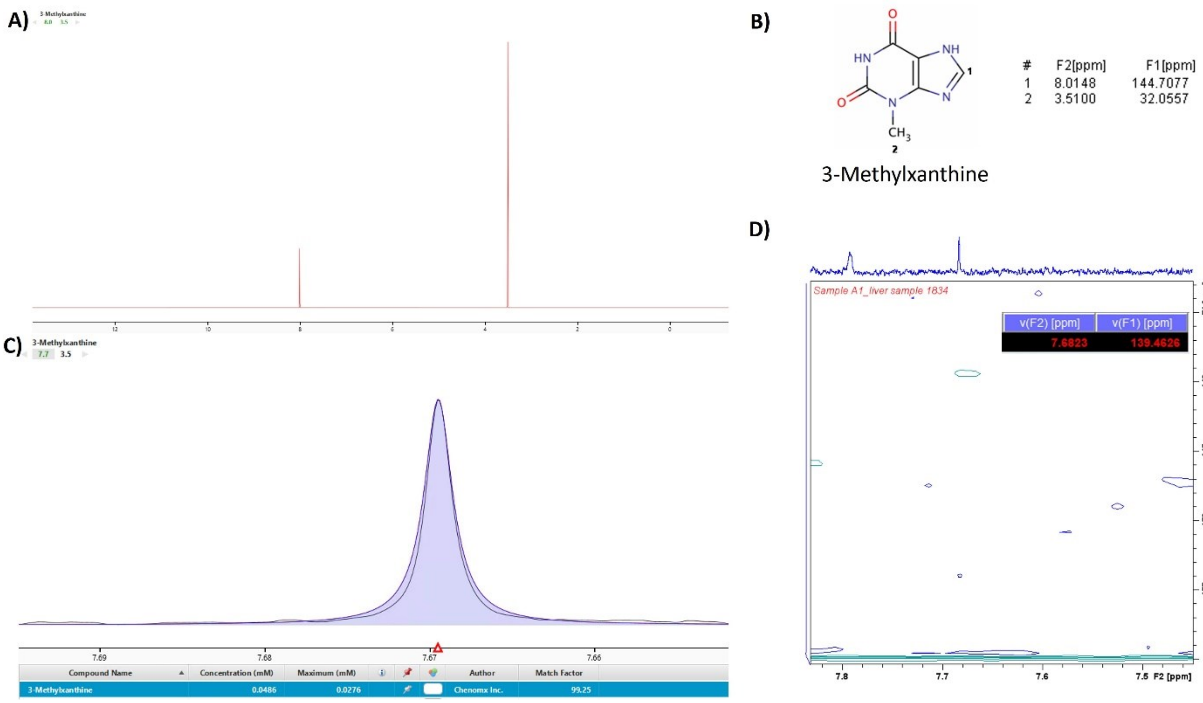
A)
3-Methylxanthine
8.0 3.5
B)
F2[ppm] F1[ppm]
1 8.0148 144.7077
2 3.5100 32.0557
3-Methylxanthine
C)
3-Methylxanthine
7.7 3.5
Compound Name
Concentration (mM)
Maximum (mM)
Author
Match Factor
3-Methylxanthine 0.0486 0.0276 Chenomx Inc. 99.25
D)
Sample A1_liver sample 1834
v(F2) [ppm] v(F1) [ppm]
7.6823 139.4626
F1 [ppm]
F2 [ppm]

Figure S9

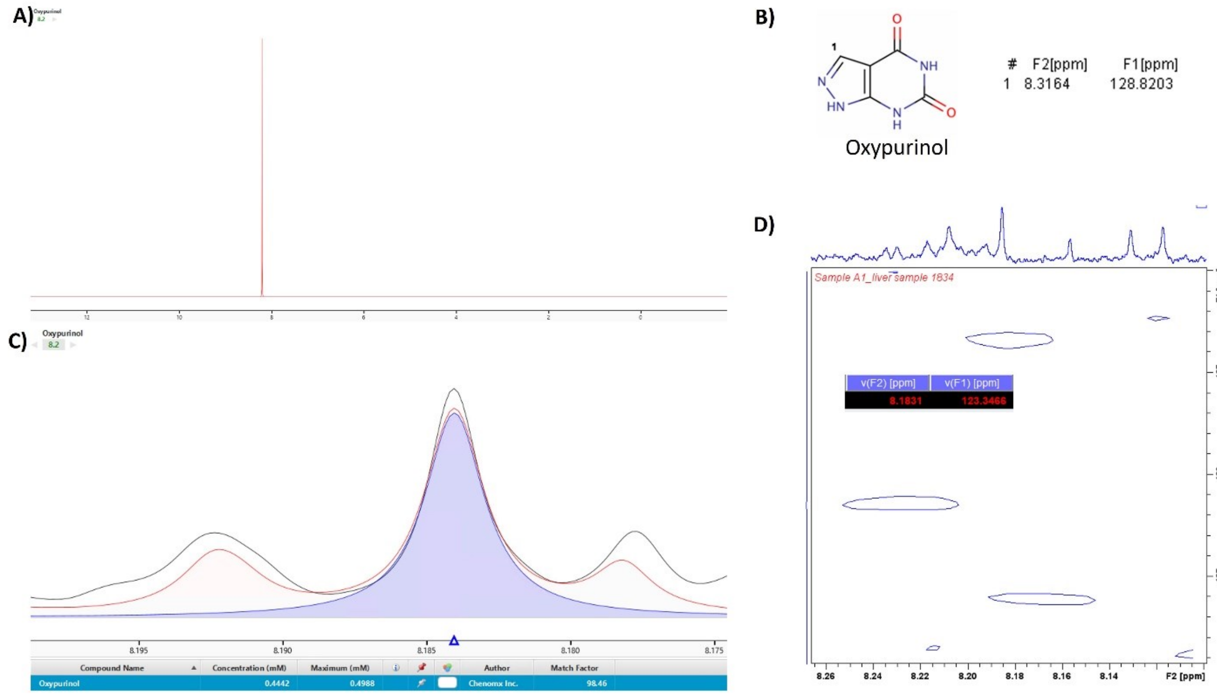
A)
Oxypurinol
B)
Oxypurinol
F2[ppm] F1[ppm]
1 8.3164 128.8203
C)
Oxypurinol
8.195
8.190
8.185
8.180
8.175
Compound Name
Concentration (mM)
Maximum (mM)
Author
Match Factor
Oxypurinol
0.4442
0.4988
Chenomx Inc.
98.46
D)
Sample A1_liver sample 1834
v(F2) [ppm]
v(F1) [ppm]
8.1831
123.3466
F1 [ppm]
125
130
135
8.26
8.24
8.22
8.20
8.18
8.16
8.14
F2 [ppm]

Figure S10

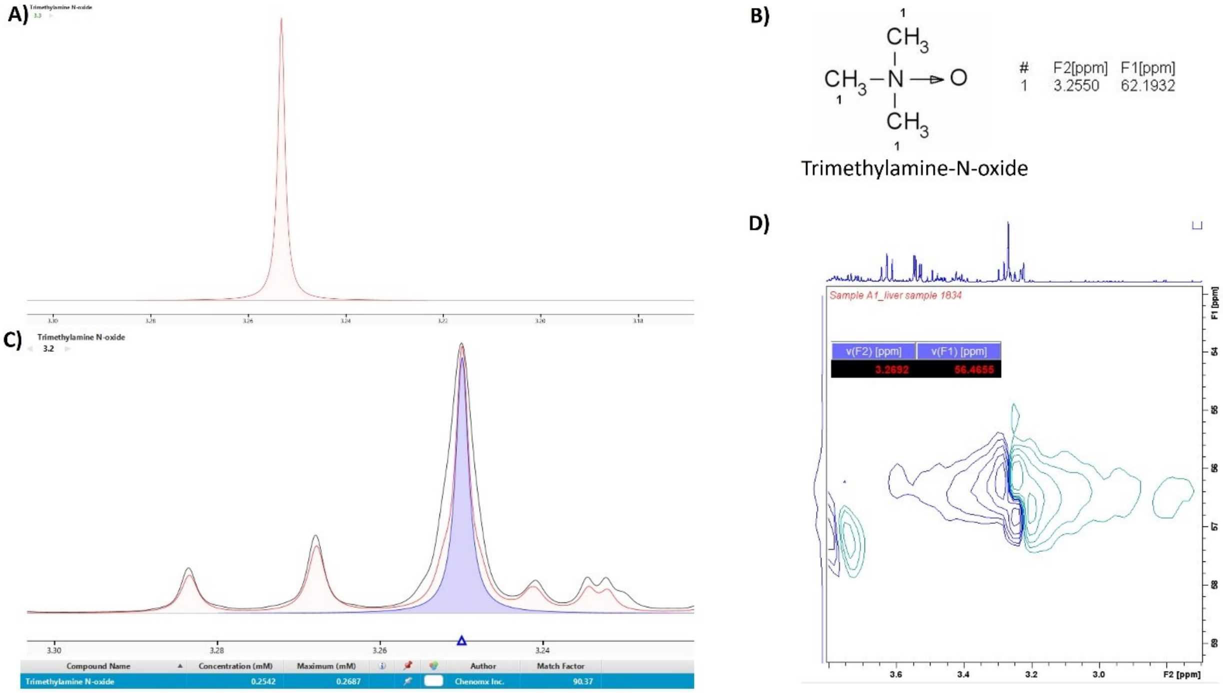
A)
Trimethylamine N-oxide
B)
Trimethylamine-N-oxide
F2[ppm] F1[ppm]
1 3.2550 62.1932
C)
Trimethylamine N-oxide
3.2
Compound Name
Concentration (mM)
Maximum (mM)
Author
Match Factor
Trimethylamine N-oxide
0.2542
0.2687
Chenomx Inc.
90.37
D)
Sample A1_liver sample 1834
v(F2) [ppm]
v(F1) [ppm]
3.2692
56.4655
F1 [ppm]
F2 [ppm]
